# BLH3 Regulates the ABA Pathway and Lignin Synthesis Under Salt Stress in *Lilium pumilum*

**DOI:** 10.3390/plants14121860

**Published:** 2025-06-17

**Authors:** Wenhao Wan, Lingshu Zhang, Xingyu Liu, Huitao Cui, Miaoxin Shi, Hao Sun, Wei Yang, Xinran Wang, Fengshan Yang, Shumei Jin

**Affiliations:** 1 Key Laboratory of Saline-Alkali Vegetation Ecology Restoration, College of Life Sciences, Northeast Forestry University, Ministry of Education, Harbin 150028, China; wwh13589976069@163.com (W.W.); 15942937576@163.com (L.Z.); nku1dsa@163.com (X.L.); meow0198@163.com (H.C.); shimiaoxin0224@163.com (M.S.); hao870882@163.com (H.S.); 2 Heilongjiang Agricultural Technology Extension Station, Harbin 150090, China; yxwyyy@126.com; 3 Faculty of Humanities, The Hong Kong Polytechnic University, Hong Kong SAR, China; 22102991d@connect.polyu.hk; 4 Engineering Research Center of Agricultural Microbiology Technology, Ministry of Education, Heilongjiang University, Harbin 150080, China; 5 Heilongjiang Provincial Key Laboratory of Ecological Restoration and Resource Utilization for Cold Region, Heilongjiang University, Harbin 150080, China; 6 Key Laboratory of Molecular Biology, College of Heilongjiang Province, College of Life Sciences, Heilongjiang University, Harbin 150080, China

**Keywords:** ABA pathway, *BLH3*, *Lilium pumilum*, lignin content, salt stress

## Abstract

BEL1-like homeodomain protein 3 (BLH3) plays a crucial role in plant development. However, its involvement in the salt stress response has not been studied. In this study, we investigated the molecular mechanism underlying the response of *LpBLH3* to salt stress in *Lilium pumilum* (*L. pumilum*) using various techniques, including quantitative PCR (RT-qPCR), determination of physiological indices of plant after Saline-Alkali stress, yeast two-hybrid screening, luciferase complementation imaging (LCI), and chromosome walking to obtain the promoter sequence, analyzed by PlantCARE, electrophoretic mobility shift assay (EMSA), and then dual-luciferase reporter assay(LUC). RT-qPCR analysis revealed that *LpBLH3* is most highly expressed in the leaves of *L. pumilum*. The expression of *LpBLH3* peaks at 24 or 36 h in the leaves under different saline stress. Under various treatments, compared to the wild type (WT), the *LpBLH3* overexpression lines exhibited less chlorosis and leaf curling and stronger photosynthesis. The overexpression of *LpBLH3* can enhance lignin accumulation in root and stem by positively modulating the expression of crucial genes within the lignin biosynthesis pathway. Y2H and LCI analyses demonstrated that LpBLH3 interacts with LpKNAT3. Additionally, EMSA and LUC analyses confirmed that LpBLH3 can bind to the promoter of *LpABI5* and upregulate the expression of *ABI5* downstream genes (*LpCAT1*/*LpATEM*/*LpRD29B*). In summary, LpBLH3 enhances the plant’s salt tolerance through the ABA pathway and lignin synthesis. This study can enrich the functional network of the BLH transcription factor family, obtain *Lilium pumilum* lines with good saline-alkali resistance, expand the planting area of *Lilium pumilum*, and improve its medicinal and ornamental values. Additionally, the functional analysis of the BLH transcription factor family provides new insights into how crops adapt to the extreme growth environment of saline-alkali soils.

## 1. Introduction

The western part of the Songnen Plain is one of the three major concentrated distribution areas of soda saline-alkaline soil in the world, with an area of saline-alkali land exceeding 300 million hm^2^ [1]. The increasingly severe soil salinization hinders the growth and progress of plants. Salt stress is one of the important abiotic stresses restricting crop growth and yield, which inhibits crop physiological metabolism through multiple mechanisms such as osmotic stress, ion toxicity, and oxidative damage [2]. Plants have evolved a variety of precise regulatory mechanisms through long-term adaptation to respond to saline-alkali stress. According to current research results, the main mechanisms of salt-alkali tolerance can be summarized into three aspects: osmotic regulation, ion regulation, and reactive oxygen species (ROS) scavenging regulation [3]. Osmotic regulation maintains intracellular osmotic balance by regulating hydrophilic inorganic ions and organic compatible solutes in cells [4,5]. Key organic compatible solutes such as proline, soluble sugars, and soluble proteins can enhance plant stress tolerance. ROS scavenging regulation mediates ROS elimination, as well as control and repair of cell damage, strictly regulating ROS concentration to ensure the balance of plant somatic metabolism [6]. In this study, *Lilium pumilum* not only has extremely high ornamental value, but also possesses excellent characteristics such as strong saline-alkaline resistance [7]. Therefore, *L. pumilum* is one of the most important materials for studying the saline-alkaline tolerance of plants.

The TALE (Three Amino Acid Loop Extension) homeobox genes can be classified into two subfamilies: KNOTTED-like homeodomain (KNOX) and BEL1-like homeodomain (BLH) [8]. The BLH sub-family is characterized by a conserved N-terminal SKY domain and a BELL domain preceding the homeobox domain. The SKY and BELL domains together form the POX domain, which can interact with the MEINOX domain of KNOX family proteins, generating homodimers or heterodimers involved in regulating plant growth and stress responses [9,10]. The high conservation of these two functional domains is crucial for the proper functioning of plant BLH family proteins [11]. BLH homologous proteins have been demonstrated to control meristem formation or maintenance, organ morphogenesis, organ positioning, and several aspects of the reproductive phase. Studies in the field have indicated that members of the TALE gene family not only participate in hormone regulatory pathways but also respond to various abiotic stress processes [12]. In *Arabidopsis thaliana*, the BLH family consists of 12 genes, including *BLH1* to *BLH10*. Among them, genes such as *BLH2*, *BLH4*, and *BLH6* are involved in plant morphogenesis [13]. BLH gene family participate in the entire plant growth process, with *BLH3* contributing to meristem formation and maintenance. BLH3 can form a dimeric structure with the STM to regulate floral organ development [14]. Simultaneously, BLH3 interacts with OFP1 to modulate inflorescence architecture and flowering [15]. In *Gossypium hirsutum*, GhBLH5-A05 can interact with GhKNAT6-A03 to promote the expression of drought stress-responsive genes *GhRD20-A09* and *GhDREB2C-D05*, thereby enhancing the tolerance of cotton to drought stress [16,17]. When the *GmBLH4* gene is overexpressed in *Arabidopsis thaliana*, the transgenic *Arabidopsis thaliana* shows significantly higher seed vigor and germination rate than the WT under high-temperature and high-humidity stress. GmBLH4 interacts with GmSBH1 to form a complex, which jointly regulates the response of soybean to high-temperature and high-humidity stress [8]. In *Toona sinensis*, *TsBLH4* enhances the plant’s tolerance to osmotic stress by interacting with TsKNOX6 [18]. The seven BLH family genes in *populus* could respond to salt stress [19]. In *Arabidopsis thaliana*, compared to the wild type (WT), overexpressing *GmBLH4* lines exhibited remarkably improved tolerance to salt and humidity (HTH) stress [8]. Since the research on the BLH gene family in response to salt stress is extremely limited, we explored whether *LpBLH3* can confer salt stress tolerance to *L. pumilum*.

Lignin, a key component of the cell wall, is central to the biosynthesis of secondary cell walls [20]. In comparison to normal plant lines, salt-tolerant lines typically display increased lignin content and thickened cell walls, highlighting the importance of cell wall fortification in plant adaptation to salt stress [21]. In *Arabidopsis thaliana*, the accumulation amount of lignin can impact the structure of the secondary cell wall and the plant’s response to saline stress. BLH family genes consistently show associations with lignin biosynthesis. In *Arabidopsis thaliana*, *BLH2*, *BLH3*, and *BLH6* have been demonstrated to be involved in cell wall development [11]. In *Gossypium hirsutum*, the *GhBLH6* gene participates in regulating secondary cell wall development. *AtBLH2*, *AtBLH3*, *AtBLH6*, and *AtBLH10* are closely related to cell wall synthesis and cell wall components [21,22]. In *Camellia japonica*, CcBLH6 functions as a positive regulator in lignin biosynthesis during the lignification stage of camellia fruits [23]. Thus, we hypothesize that LpBLH3 might be conferring plant tolerance to salt stress by increasing lignin accumulation and leading to thickening of the cell wall. The interaction between the BLH and KNAT family produce homodimers or heterodimers that govern plant growth and stress responses [24]. In *Arabidopsis thaliana*, *KNAT3* regulates lignin biosynthesis, promotes the production of secondary cell walls in vessels, and provides mechanical support for the stems, BLH1 collaborated with KNAT3 enhancing the retention of KNAT3 in the nucleus [25,26]. Therefore, we hypothesize that LpBLH3 can interact with LpKNAT3 to regulate lignin biosynthesis.

The BLH family is closely related to the ABA pathway [11,27,28]. To further reveal the mechanism by which LpBLH3 impacts plant salt tolerance, we researched its potential regulation of ABA pathway. In *Arabidopsis thaliana*, BLH1 and KNAT3 collaborate to enhance ABA responses by triggering the activation of the *ABI3* promoter through the TGGA motif [29]. In *Gossypium hirsutum*, GhBLH1 can recognize and bind to the TGGA motif of *ABI3* and in response to salt stress [30,31]. This activation of *ABI3* may further influence the downstream events in the ABA signaling pathway where *ABI5* is also involved. ABI5 belongs to the basic leucine zipper (bZIP) transcription factor family, it plays a regulatory role under high salinity conditions by regulating the expression of genes that contain abscisic acid response elements (ABREs) in their promoter regions [32,33,34,35]. In *Arabidopsis thaliana*, *ABI5* can participate in the plant’s response to ABA and stress resistances by regulating downstream genes. ABI5 binds to the promoter of *CAT1* and activates the expression of *CAT1*, and it enhances the plant’s ability to scavenge reactive oxygen species and strengthens the plant’s tolerance to salt stress [36]. ABI5 can bind to specific cis-acting elements in the promoter region of the *AtATEM* gene, promoting the expression of the AtATEM protein, which in turn protects cells from the harm of adverse conditions such as high salinity [37]. ABI5 can bind to the promoter of the *AtRD29B* gene, regulate its transcription, and enhance the plant’s tolerance to stresses such as salt and drought [38]. Therefore, we verified whether BLH3 regulates the expression of *ABI5* and its downstream genes in response to saline-alkaline stress by binding to the *ABI5* promoter.

Previous studies have mostly focused on the role of BLH3 in growth and development. In this study, we investigates the hypothesis that LpBLH3 plays a role in regulating the ABA signaling pathway and lignin biosynthesis pathway during abiotic stress responses in plants. Additionally, we explore the possibility that LpBLH3 interacts with LpKNAT3 to coordinate the expression of stress-responsive genes. Our research not only elucidates how plants orchestrate gene expression during stress but also opens up new avenues for developing crops with improved resilience to abiotic stresses.

## 2. Results

### 2.1. Cloning and Bioinformatic Analysis of the LpBLH3 Gene

Sequencing results revealed that the open reading frame (ORF) of the *LpBLH3* gene is 1800 bp in length, encoding a protein of 599 amino acids. Amino acid sequence alignment showed a high degree of homology to BLH3 proteins from other plants (Figure S1). The LpBLH3 protein contains two conserved domains: a POX domain, located between amino acids 306–459, and a HOX (HD) domain, located between amino acids 502–566 (Figure S2). The phylogenetic analysis indicated that LpBLH3 is most closely related to the BLH3 protein from *Asparagus officinalis* (Figure S3).

### 2.2. Analysis of LpBLH3 Expression

**Figure 1:**
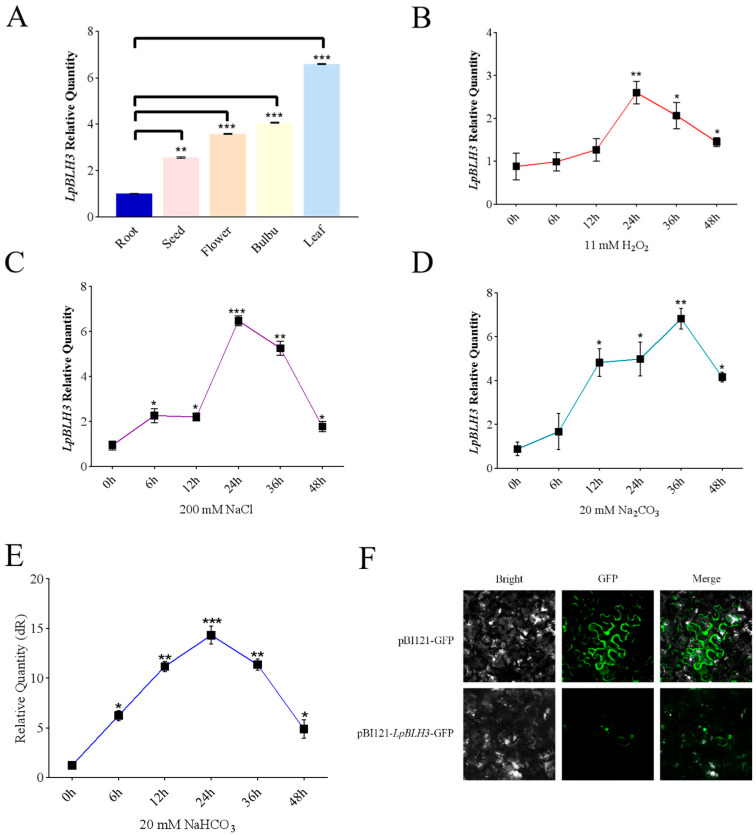
Expression and subcellular localization of LpBLH3. (**A**) Expression of *LpBLH3* in the root, bulb, leaf, flower, and seed of wild-type *Lilium pumilum* plants. cDNA was obtained from the root, bulbus, leaf, flower, and seed of *L. pumilum*, and the expression levels of *LpBLH3* were quantified using Real-Time Quantitative PCR. CK (No treatment) was used as a control. Asterisks (**) and (***) indicate statistically significant differences at *p* < 0.01 and *p* < 0.001, respectively. Data are presented as mean ± SD from three replicates. (**B**) Real-Time Quantitative PCR analysis of *LpBLH3* expression in *L. pumilum* under 11 mM H_2_O_2_ treatment for 6 h, 12 h, 24 h, 36 h, and 48 h. CK (No treatment) was used as a control. Asterisks (*) and (**) indicate statistically significant differences at *p* < 0.05 and *p* < 0.01, respectively. Data are presented as mean ± SD from three replicates. (**C**) Real-Time Quantitative PCR analysis of *LpBLH3* expression in *L. pumilum* under 200 mM NaCl treatment for 6 h, 12 h, 24 h, 36 h, and 48 h. CK (No treatment) was used as a control. Asterisks (*) (**) and (***) indicate statistically significant differences at *p* < 0.05, *p* < 0.01 and *p* < 0.001, respectively. Data are presented as mean ± SD from three replicates. (**D**) Real-Time Quantitative PCR analysis of *LpBLH3* expression in *L. pumilum* under 20 mM Na_2_CO_3_ treatment for 6 h, 12 h, 24 h, 36 h, and 48 h. CK (No treatment) was used as a control. Asterisks (*) and (**) indicate statistically significant differences at *p* < 0.05 and *p* < 0.01, respectively. Data are presented as mean ± SD from three replicates. (**E**) Real-Time Quantitative PCR analysis of *LpBLH3* expression in *L. pumilum* under 20 mM NaHCO_3_ treatment for 6 h, 12 h, 24 h, 36 h, and 48 h. CK (No treatment) was used as a control. Asterisks (*) (**) and (***) indicate statistically significant differences at *p* < 0.05, *p* < 0.01 and *p* < 0.001, respectively. Data are presented as mean ± SD from three replicates. (**F**) Subcellular localization of LpBLH3 protein. Green fluorescence indicates GFP expression. pBI121-GFP and pBI121-*LpBLH3*-GFP constructs were transiently transformed into *N. benthamiana* cells using a biolistic transformation method. Samples were examined under a microscope equipped with a fluorescence module. Scale bar = 100 μm.

To explore the expression levels of *LpBLH3* in different organs, RT-qPCR analysis showed that *LpBLH3* expression was highest in the leaf, approximately 7-fold higher than in the root (Figure 1A). Under stress conditions with 11 mM H_2_O_2_, 200 mM NaCl, 20 mM Na_2_CO_3_, or 20 mM NaHCO_3_, *LpBLH3* expression in *L. pumilum* leaf significantly increased, peaking at 24 h or 36 h, and gradually decreased with longer treatment times (Figure 1B–E).

These results indicate that *LpBLH3* is highly responsive to saline stresses. Subcellular localization analysis using a Carl Zeiss fluorescence microscope detected green fluorescence in the nucleus (Figure 1F), suggesting that LpBLH3 protein is localized in the nucleus, consistent with its role as a transcription factor.

### 2.3. Generation of LpBLH3 Over-Expressing Lines and Measure of Physiological Indexes

RT-qPCR was used to measure the expression levels of *LpBLH3* in the WT and overexpression lines. The overexpression lines #1–#8 exhibited higher expression levels of *LpBLH3* compared to the WT. The three *L. pumilum* lines with the highest expression levels (#6, #7, #8) were selected for further experiments (Figure S4).

**Figure 2:**
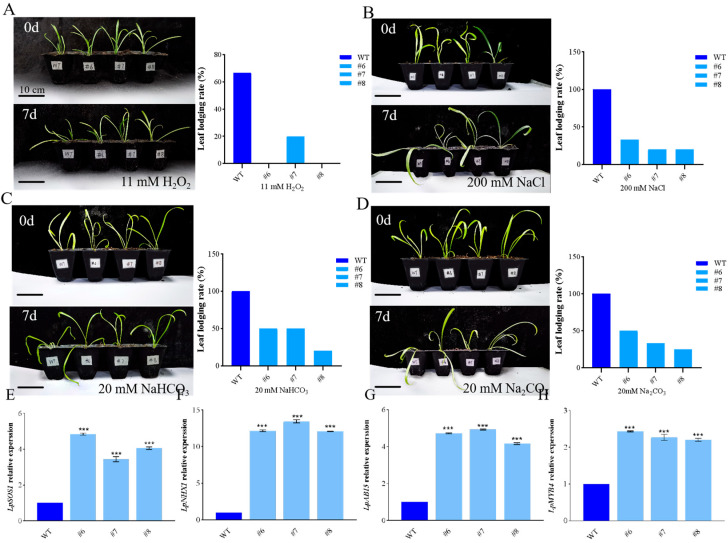
Phenotypes of transgenic *L. pumilum* under saline-alkaline stress. (**A**–**D**) Plant growth phenotype and leaf lodging rate (%) of wild-type (WT) and *LpBLH3* overexpressing *L. pumilum* lines were evaluated. Plants were grown on the same medium supplemented with 11 mM H_2_O_2_, 200 mM NaCl, 20 mM Na_2_CO_3_, and 20 mM NaHCO_3_ for 0 and 7 days. WT: wild-type; #6, #7, #8: *LpBLH3* overexpressing lines. Scale bar = 10 cm. (**E**–**H**) Determination of relative expression of genes related to salt-alkali stress (*LpSOS1*/*LpNHX1*/*LpABI5*/*LpMYB4*). WT: wild type. #6, #7 and #8: *LpBLH3* overexpressing lines. Note: *** *p* < 0.001, standard error of three biological replicates.

In order to observe the effect of *LpBLH3* overexpression on improving plant salt tolerance, the WT and overexpression lines were subjected to stress treatments with 11 mM H_2_O_2_, 200 mM NaCl, 20 mM Na_2_CO_3_, or 20 mM NaHCO_3_. Under normal conditions, there were no significant differences in growth between the WT and *LpBLH3* overexpressing lines. However, under four stress treatments, the leaves of *LpBLH3* overexpressing lines remained mostly green with minimal wilting and yellowing. In contrast, the wild-type plants showed more severe wilting. The majority of the overexpressing lines remained upright with a low leaf lodging rate, while the WT exhibited significant wilting (Figure 2A–D). Under four stress treatments, the leaves lodging rate of *LpBLH3* overexpression plants was approximately 50% lower than that of the WT. After 24 h of treatment with 20 mM NaHCO_3_, the expression levels of stress-related genes *LpABI5*, *LpSOS1*, *LpNHX1*, and *LpMYB4* were found to be higher in the three *LpBLH3* overexpressing lines compared to the WT (Figure 2E–H). These results indicate that *LpBLH3* overexpression positively responds to salt stress.

**Figure 3:**
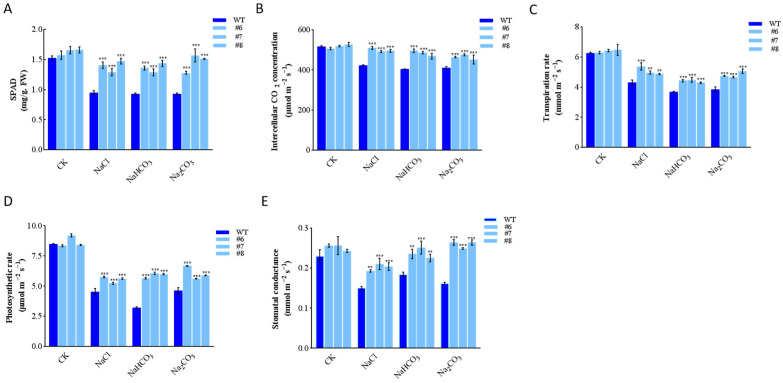
Determination of physiological indexes of *LpBLH3* over-experssing *L. pumilum* under saline stress. (**A**) Chlorophyll content. (**B**) Intercellular CO_2_ concentration. (**C**) Transpiration rate. (**D**) Photosynthetic rate. (**E**) Stomatal conductance. WT: wild type. #6; #7; #8: three selected *LpBLH3* overexpressing lines with high expression level. ** *p* < 0.01 and *** *p* < 0.001 standard error of three biological replicates.

We detected and analyzed the physiological indexes. The measurement results of chlorophyll content, stomatal conductance, transpiration rate, net photosynthetic rate, and intercellular CO_2_ concentration showed a significant decrease in WT. However, in the three *LpBLH3* overexpressing lines, the chlorophyll content were approximately 1.5 times higher than the WT. The stomatal conductance were approximately 1 times higher than the WT, and the intercellular CO_2_ concentration were approximately 1.25 times higher than the WT. While photosynthetic and transpiration rates still decreased, the amount reduction was not obvious in the *LpBLH3* overexpressing lines compared to the WT. After exposure to the four stress treatments, the photosynthetic indexes in the *LpBLH3* overexpressing lines were superior to those in the WT (Figure 3A–E).

Compared to the WT, *LpBLH3* overexpressing lines exhibited higher proline content and lower MDA levels (Figure S5A,B), which enhanced cell water retention and enzyme activity.

### 2.4. The Effect of the LpBLH3 Gene on Lignin Content

**Figure 4:**
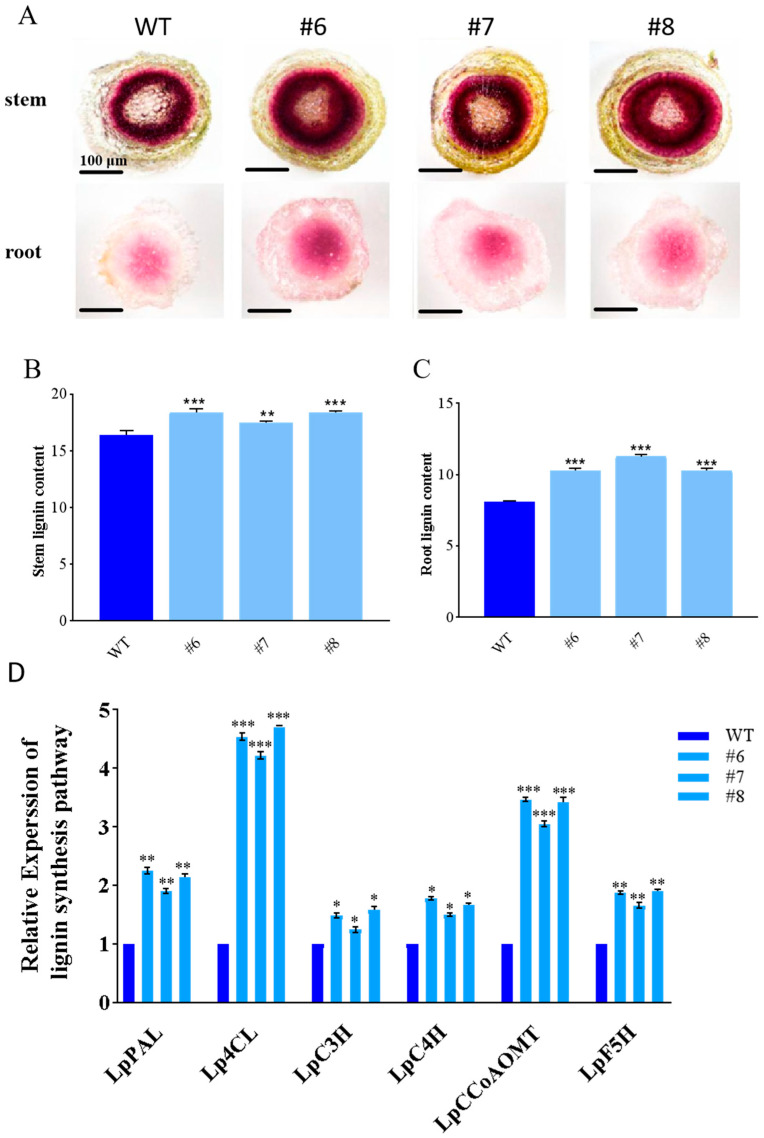
Analysis of gene expression in lignin synthesis pathway. (**A**) Phloroglucinol staining of *L. pumilum* from detached stem and the root. WT: wild type. #6, #7 and #8: *LpBLH3* overexpressing lines. The depth of staining reflects the amount of lignin accumulation in the cells. Standard error of three biological replicates. Scale bar = 100 μm. (**B**,**C**) Lignin content of stem and root. WT: wild type. #6, #7, #8: *LpBLH3* overexpressing lines. Note: ** *p* < 0.01 and *** *p* < 0.001, standard error of three biological replicates. (**D**) Analysis of gene expression in lignin synthesis pathway. (*LpPAL*/*Lp4CL*/*LpC3H*/*LpC4H*/*LpCCoAOMT*/*LpF5H*). WT: wild type. #6, #7, #8: *LpBLH3* overexpressing lines. Note: * *p* < 0.05, ** *p* < 0.01, *** *p* < 0.001, standard error of three biological replicates.

After 20 mM NaHCO_3_ stress for 24 h, phloroglucinol staining was conducted on the stem and root of both WT and *LpBLH3* overexpressing lines. The staining results of the stem cross-sections clearly showed that, compared to the WT, the lignin-rich areas in the stem cross-sections of the *LpBLH3* overexpressing lines were significantly wider and displayed a darker color. Similarly, in the root cross-sections, a notable difference in lignin accumulation was observed between the *LpBLH3* overexpressing lines and the WT (Figure 4A). The measurement results of lignin showed that lignin accumulation of *LpBLH3* expression lines were approximately 1.57 times higher than WT in root (Figure 4B,C). This suggests a positive correlation between *LpBLH3* expression, lignin accumulation, and the plant’s ability to withstand saline-alkaline conditions.

The expression levels of key genes involved in the lignin synthesis pathway, including *LpPAL*, *Lp4CL*, *LpC3H*, *LpC4H*, *LpCCoAOMT*, and *LpF5H*, were examined. After treating both the *LpBLH3* overexpressing lines and the WT lines with 20 mM NaHCO_3_ for 24 h, it was found that the expression levels of these genes were significantly higher in the three *LpBLH3* overexpressing lines compared to the WT (Figure 4D). These findings demonstrate that the overexpression of *LpBLH3* positively regulates the expression of crucial genes in the lignin biosynthesis pathway. As a result, it promotes lignin accumulation in both the roots and stems of the plants, contributing to their enhanced resistance to saline-alkaline stress.

### 2.5. Analysis of LpBLH3-Interacting Proteins

**Figure 5:**
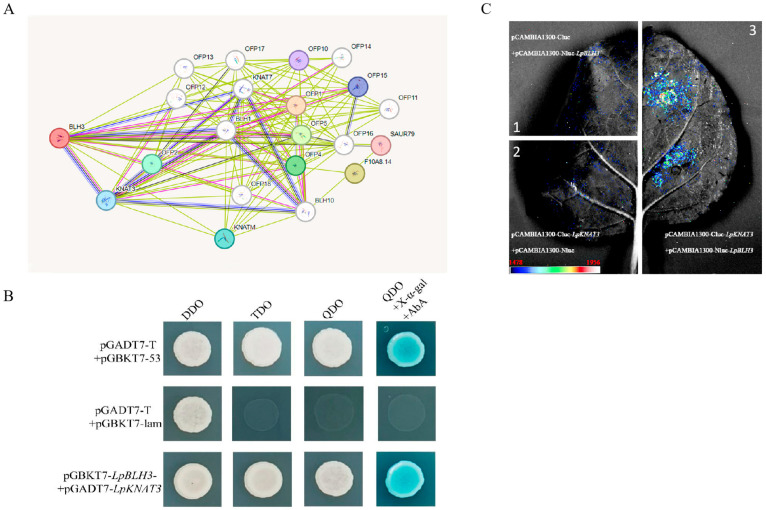
Validation of the interaction between LpBLH3 and LpKNAT3. (**A**) Prediction of interaction protein of LpBLH3 based on STRING. (**B**) Yeast two-hybrid assay to verify the relationship between LpBLH3 and LpKNAT3. The cotransformation of pGADT7 + pGBKT7, pGADT7 + pGBKT7-*LpBLH3*, and pGBKT7 + pGADT7-*LpKNTA3* were used as controls. Only pGADT7-*LpKNTA3* and pGBKT7-*LpBLH3* co-transformed colonies turned blue on SD/-Trp/-Leu/-His/-Ade + X-α-gal medium. (**C**) LCI assay to discover the relationship between LpBLH3 and LpKNAT3 were co-injected into *N. benthamiana* cells. (**1**) NLUC + CLUC; (**2**) *LpKNAT3*-CLUC + NLUC; (**3**) *LpBLH3*-NLUC + *LpKNAT3*-CLUC were used as controls. Scale bar = 1 cm.

The plasmids of *LpBLH3*-pGBKT7 was transformed into Y2H Gold yeast strains, and a yeast two-hybrid screen identified ten different full-length or partial interacting protein, including KNAT3 (Table S5). Prediction of LpBLH3 interacting proteins revealed that the candidate proteins included KNAT3 (Figure 5A).

Previous studies have shown that KNAT3 is involved in responses to abiotic stress and lignin bio-accumulation. Therefore, we selected LpKNAT3 as a candidate interacting protein for LpBLH3.

Both the control and experimental groups grew normally on SD/-Trp-Leu medium. Co-transformation of pGBKT7-*LpBLH3* and pGADT7-*LpKNAT3* into Y2H Gold yeast strains enabled growth on SD/QDO + X-α-gal + AbA medium, and the colonies turned blue, similar to the positive control (Figure 5B). This result confirmed that LpBLH3 and LpKNAT3 proteins interact in the yeast two-hybrid system. Additionally, LCI analysis further validated the interaction between LpBLH3 and LpKNAT3 proteins, as fluorescence signals were observed only in tobacco leaves co-infiltrated with pBS-35S: *LpBLH3*-VN154 and pBS-35S: *LpKNAT3*-VC80 constructs (Figure 5C).

### 2.6. BLH3 Regulates the ABI5 Expression

**Figure 6:**
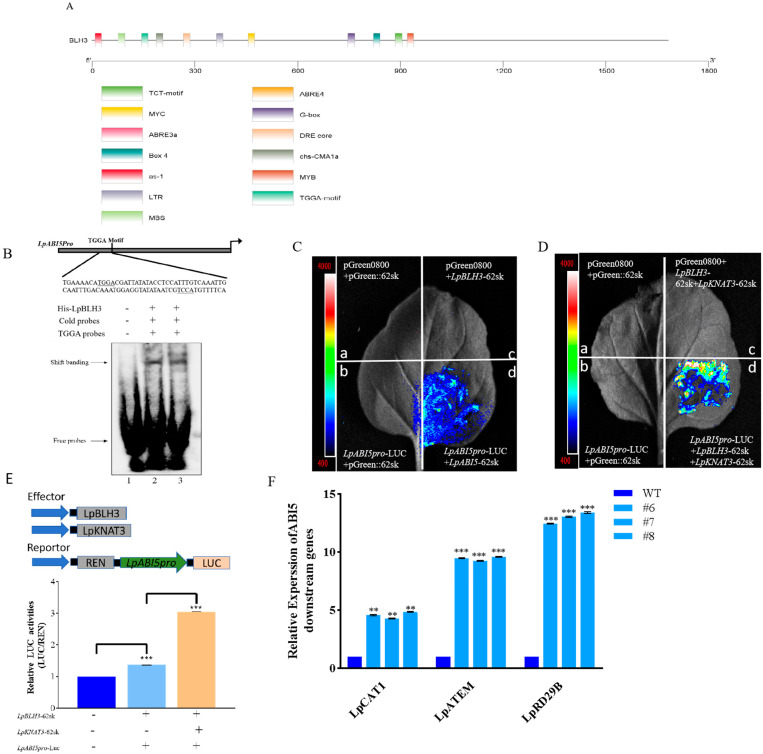
BLH3 regulates the *ABI5* expression. (**A**) Visual analysis of the *LpABI5* promoter. The cis-acting element analysis of the *LpABI5* promoter sequence was carried out using the PlantCARE website. The obtained promoter sequences were visualized and analyzed using TBtools software. (**B**) Schematic diagram of the *ABI5* promoter showing BLH3-binding TGGA motifs. Binding affinity of LpBLH3 to the *ABI5* promoter was evaluated using EMSA. The first, second and third track represents control, normal, and competitive, respectively. (**C**) Dual-luciferase reporter assay. (a) LUC +62SK, (b) *LpABI5*-pro-LUC + 62SK, (c) *LpBLH3*-62SK + LUC, (d) *LpBLH3*-62SK + *LpABI5*-pro-LUC. Empty vector 62SK + LUC was used as a negative control. Scale bar = 1 cm. (**D**) Dual-luciferase reporter assay. (a) LUC +62SK, (b) *LpABI5*-pro-LUC + 62SK, (c) *LpBLH3*-62SK + *LpKNAT3*-62SK + LUC, (d) *LpBLH3*-62SK + *LpKNAT3*-62SK + *LpABI5*-pro-LUC. Empty vector 62SK + LUC were used as a negative control. Scale bar = 1 cm. (**E**) Quantification was performed by normalizing firefly luciferase (LUC) activity to the activity of Renilla luciferase (REN), and 35S: REN was used as the internal control. Relative luciferase activities were determined using *LpBLH3*-pGreenII62-SK and *LpKNAT3*-pGreenII62-SK as the effector compared with the control effector (pGreenII62-SK empty vector). Values are means SD. (**F**) Analysis the expression levels of the *LpABI5* downstream genes (*LpCAT1*/*LpATEM*/*LpRD29B*). WT: wild type. #6, #7, #8: *LpBLH3* overexpressing lines. Note: ** *p* < 0.01 and *** *p* < 0.001, standard error of three biological replicates.

To gain a comprehensive understanding of the regulatory elements associated with *LpABI5*, a series of experiments were carried out. An 852 bp upstream promoter sequence of *LpABI5* was successfully isolated through the application of chromosome walking PCR (the complete sequence of the *LpABI5* promoter is presented in Table S3). The potential cis-acting elements within the *LpABI5* promoter was analyzed by the PlantCARE tool (Table S4). A visual exploration of the promoter sequence was performed using TBtools software (Figure 6A). Through this visual analysis, multiple recognition sites (TGGA) specific to the BLH3 transcription factor were uncovered, demonstrating that the LpBLH3 protein can bind to the promoter of *LpABI5*.

To validate this hypothesis, an in vitro EMSA was implemented. Firstly, the optimal conditions for inducing LpBLH3 protein expression were determined to be an OD_600_ of 0.6, with the addition of 0.5 mM IPTG, followed by incubation at 37 °C with shaking at 240 rpm for 4 h. In SDS-PAGE analysis, the induction of protein expression was confirmed by the presence of distinct bands around 72 kDa at 1, 2, 3, and 4 h post-induction (Figure S6A). The LpBLH3 protein was then purified using BeyoGold™ His-tag Purification Resin Ni-NTA affinity chromatography (Figure S6B). Further validation of LpBLH3 binding to the *LpABI5* promoter was performed using EMSA, where the interaction between LpBLH3 protein and a biotin-labeled probe resulted in a shifted band, which was inhibited by the addition of a competitive probe (Figure 6B). These results demonstrate that LpBLH3 directly bind to *LpABI5* promoter.

To verify whether the interaction between KNAT3 and BLH3 affects the expression of *ABI5*, we conducted a Dual-luciferase reporter assay. When *LpBLH3*-62SK, *LpKNAT3*-62SK, and *LpABI5Pro*-LUC were co-injected, the LUC signal was substantially stronger than that observed in the leaves co-infiltrated with just *LpBLH3*-62SK and *LpABI5*Pro-LUC (Figure 6C,D). And the luciferase activity was higher. (Figure 6E). Taken together, these experimental findings establish that LpKNAT3 facilitates LpBLH3-mediated regulation of *LpABI5* expression.

Both *LpBLH3* overexpressing lines and WT lines were subjected to a 24 h treatment with 20 mM NaHCO_3_. The expression levels of the downstream genes of *LpABI5*, namely *LpCAT1*, *LpATEM*, and *LpRD29B*, were carefully quantified. Intriguingly, the expression levels of these genes were found to be considerably higher in the three *LpBLH3* overexpressing lines compared to the WT (Figure 6F). These results demonstrate that LpBLH3 can activate the *ABI5* promoter, and LpABI5 then acts as a key regulator, positively influencing the expression of downstream genes in the ABA pathway. This regulatory cascade potentially equipped the plant with enhanced stress tolerance capabilities.

## 3. Discussion

In this study, *LpBLH3* was cloned from *L. pumilum*. The LpBLH3 protein was found to possess a HOX domain spanning amino acids 502 to 566 in other plants (Figures S1–S3). In previous laboratory investigations, a comprehensive analysis of the transcriptome of *L. pumilum* was carried out under alkaline stress conditions. It was discovered that the expression of *LpBLH3* was significantly upregulated [39]. In this study, after short-term exposure, *LpBLH3* expression in *L. pumilum* leaf significantly increased. Although previous studies have confirmed the expression changes in *LpBLH3*, its functional mechanism—How does LpBLH3 response to salt stress—remains unclear. Current research on *BLH3* gene stress responses primarily focuses on model plants such as *Arabidopsis* and rice, with limited functional validation in woody plants like *L. pumilum*. Considering that, we investigated the functional role of *LpBLH3* in alkali stress tolerance.

To find out where *LpBLH3* functions in plants, the expression level of *LpBLH3* in different organs was analyzed by qPCR, which eventually shows that the expression of *LpBLH3* was the highest in leaves (Figure 1A). The *GmBLH4* gene expressed its maximum level after HTH stress for 168 h [8]. In this study, when the plants were treated with four different stress, *LpBLH3* expression in *L. pumilum* leaf significantly increased, peaking at 24 h or 36 h (Figure 1B–E). After 24 h of treatment with 20 mM NaHCO_3_, the expression levels of stress-related genes *LpABI5*, *LpSOS1*, *LpNHX1*, and *LpMYB4* were found to be higher in the three *LpBLH3* overexpressing lines compared to the WT (Figure 2E–H). These results indicate that *LpBLH3* might be involved in response to salt stress. Saline stress is known to inflict irreversible damage on photosynthetic organs throughout plant development, making it crucial to understand how plants mitigate such impacts. Such as, Soybean seedling growth and chlorophyll (Chl) content are reduced under NaCl stress, leading to Chl degradation [40]. Transcription factors are considered to play a crucial regulatory role in Chl degradation [41,42]. In poplar, overexpression of *PpnGRF5-1* increases leaf Chl content and regulates Chl degradation [43]. Indeed, when measuring the physiological indexes, it was found that *LpBLH3* overexpressing lines exhibited lower leaf lodging rate (Figure 2A–D), higher chlorophyll content and Photosynthetic rate (Figure 3A–E). This indicates that *LpBLH3* actively helps in reducing stress-induced damage to photosynthesis.

Lignin accumulation has been established as a critical factor in plant resistance to salt stress [44]. In comparison to normal plant lines, salt-tolerant lines typically display increased lignin content and thickened cell walls, highlighting the importance of cell wall fortification in plant adaptation to salt stress [21]. To investigate this aspect in relation to *LpBLH3*, lignin staining and content determination were performed on *LpBLH3* overexpressing lines and WT lines under 20 mM NaHCO_3_ stress. The results were conclusive: the lignin content in *LpBLH3* overexpressing lines was significantly higher than in the WT (Figure 4A–C). These data firmly suggest that overexpression of *LpBLH3* promotes lignin accumulation, thereby enhancing the salt tolerance of the plants.

The search for proteins interacting with LpBLH3 led to the identification of several candidates through yeast two-hybrid screening, with LpKNAT3 being one of them. In the plant cell context, BLH proteins frequently interact with KNOX proteins. This binding results in the formation of a heterodimer, which then translocates to the nucleus to execute its functions. Once inside the nucleus, their HD domains bind specifically to target sequences, thereby regulating the expression of downstream genes [45]. KNAT3, in particular, can interact with the POX domain of the BELL family via its MEINOX domain to form either homodimers or heterodimers, which jointly regulate plant growth and stress responses [46]. Through Y2H and luciferase complementation imaging (LCI) analyses, it was demonstrated that LpBLH3 can interact with LpKNAT3 both in vivo and in vitro (Figure 5A–C).

Previous studies have provided additional insights. The Class II KNOX genes, including *KNAT3*, *KNAT4*, *KNAT5,* and *KNAT7*, are expressed during the secondary cell wall (SCW) deposition process [47]. KNAT3 and KNAT7 act synergistically to enhance the deposition of secondary cell walls in plants [26]. Moreover, KNAT3 can interact with the key transcription factors NST1 and NST2 during secondary cell wall formation, forming a heterodimer complex that regulates F5H to promote lignin synthesis [25]. Given these findings and the fact that overexpression of *LpBLH3* increases lignin accumulation by modulating the expression of crucial genes like *LpPAL*, *Lp4CL*, *LpC3H*, *LpC4H*, *LpCCoAOMT*, and *LpF5H* within the lignin biosynthesis pathway (Figure 4D), we hypothesized that LpBLH3 and LpKNAT3 proteins might interact to jointly contribute to plant lignin synthesis and enhance salt tolerance.

To further reveal the mechanism by which LpBLH3 impacts plant salt tolerance, we researched its potential regulation of *ABI5* genes. RT-qPCR analysis revealed that the expression level of *ABI5* increased in *LpBLH3* overexpressing lines when subjected to NaHCO_3_ stress (Figure 2E–H). ABI5 encodes a member of the basic leucine zipper (bZIP) transcription factor family, which is involved in ABA signaling and plays a central role in abiotic stress responses [48]. In *Arabidopsis thaliana*, ABI5 binds to the promoter of *CAT1*, activates the expression of *CAT1*, and increases the content and activity of CAT1 protein. Therefore, it enhances the plant’s ability to scavenge reactive oxygen species (ROS), alleviates the oxidative damage caused by salt stress, and strengthens the plant’s tolerance to salt stress [36]. Employing EMSA and dual-luciferase reporter assays, we confirmed that the LpBLH3 transcription factor can specifically bind to the promoter region of *LpABI5* (Figure 6A–C). In *Arabidopsis thaliana*, ABA promotes the interaction between BLH1 and KNAT3, leading to the formation of a dimer that binds to the *ABI3* promoter via the TGGA motif, thereby enhancing the expression of *ABI3* [29]. Building on these precedents, we verified that LpBLH3 regulates the expression of *ABI5* (Figure 6D–F). Thus, we speculate that LpBLH3 may enhance the plant’s tolerance to saline stress by regulating the key gene *LpABI5* within the ABA signaling pathway.

**Figure 7:**
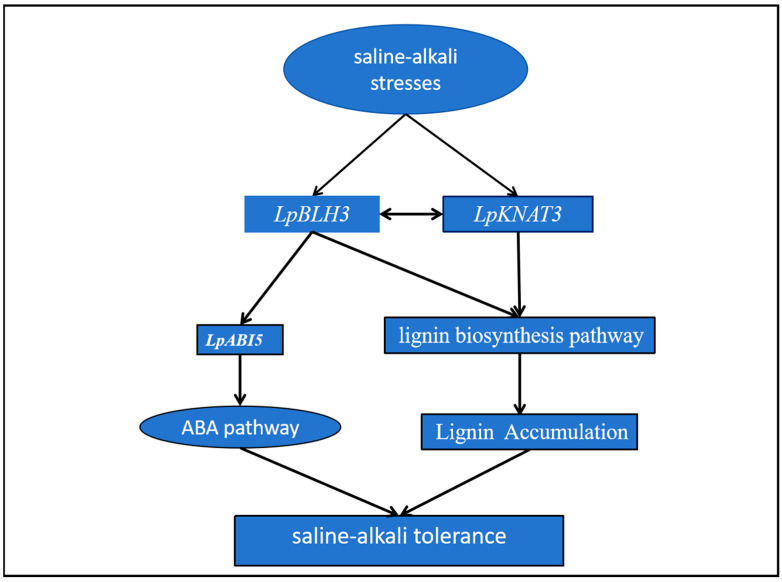
Model for the action of *LpBLH3* in conferring tolerance to saline stress in *L. pumilum*: Under saline conditions, *LpBLH3* expression increases. The model includes two main pathways through which enhances salt tolerance: ABA Pathway: LpNAT3 promotes LpBLH3 to regulate the expression of *LpABI5* by binding to the TGGA motif in the *LpABI5* promoter, activating the ABA signaling pathway to further enhance the plant’s tolerance to salt stress. Lignin Pathway: LpBLH3 may interact with LpKNAT3 to increase lignin content by positively modulating the expression of crucial genes within the lignin biosynthesis pathway, which strengthens the secondary cell wall, thereby improving the plant’s structural integrity and salt.

In summary, our research has uncovered two significant pathways by which LpBLH3 enhances plant salt tolerance. Additionally, the two pathways function independently in conferring salt stress tolerance to plants. Studies have shown that BLH3-mediated activation of the ABA signaling pathway and promotion of lignin biosynthesis are achieved through distinct regulatory mechanisms, with no significant cross-interaction at the molecular level. This independent mode of action allows plants to simultaneously perform osmotic regulation via ABA and structural reinforcement via lignin deposition, forming a multi-layered defense strategy against saline environments. Firstly, the ABA pathway plays a crucial role. BLH3 can promote the upregulation of *LpABI5* expression through its interaction with the interacting protein KNAT3, thereby triggering the expression of downstream genes in the ABA signaling cascade and participating in the ABA pathway. Secondly, the interaction between BLH3 and KNAT3 positively modulates the expression of essential genes in the lignin biosynthesis pathway. As a result, lignin accumulation is significantly increased. The augmented lignin deposition thickens the secondary cell wall, providing structural reinforcement to the plant cells. This enhanced cell wall structure acts as a physical barrier, better equipping the plant to withstand the osmotic and ionic stresses associated with high salinity environments, thereby improving the overall salt tolerance of the plant (Figure 7).

These two pathways, mediated by LpBLH3, work together to enhance the plant’s resilience to salt stress, representing a comprehensive and coordinated defense mechanism at the molecular level.

## 4. Materials and Methods

### 4.1. Plant Materials and Growth Conditions

*L. pumilum* were gathered from saline-alkali soil in Northeast China’s Daqing (46°58′ N, 125°3′ E). *Nicotiana benthamiana* were kept in the laboratory setting. All of these plants were grown in a controlled growth chamber, where the temperature was maintained at 25 ± 2 °C. The light intensity reached 2000 lux, following a 16 h light and 8 h dark photoperiod, and the relative humidity ranged from 75% to 80%.

### 4.2. Cloning and Bio-Informatics Analysis of LpBLH3 Gene

Total RNA was isolated from the leaves of *L. pumilum* by means of the OminiPlant RNA Kit (CWBIO, Beijing, China). Subsequently, cDNA was synthesized with the aid of a reverse transcription kit (Takara, Tokyo, Japan). The forward primer *LpBLH3*-F and the reverse primer *LpBLH3*-R were designed in accordance with the open reading frame (ORF) of *LpBLH3*, which was derived from the *L. pumilum* transcriptome (The primer sequences were listed in Table S1). The PCR products were then purified using the MolPure Gel Extraction Kit (Co Win Biosciences, Beijing, China), ligated into the pMD18-T vector (Takara, Tokyo, Japan), and finally transformed into *Escherichia coli* DH5α for sequencing.

The homology of LpBLH3 to other species was analyzed by BLAST in NCBI (http://www.ncbi.nlm.nih.gov, accessed on 4 May 2024). and the amino acid sequences were compared using DNAMAN software V 9.0, and the conserved domain was analyzed by CD-Search. The phylogenetic tree was constructed by MEGA7 software V 7.0.18.

### 4.3. RT-qPCR Analysis of LpBLH3 Expression

For the RT-qPCR analysis of *LpBLH3*, the primers *LpBLH3*-qPCR-F and *LpBLH3*-qPCR-R were employed, taking F-box family protein (FP) [49] and Actin (ACT) [50] as internal control genes. RNA was harvested from different tissues, namely the root, bulbus, leaf, flower, and seed of *L. pumilum*, and then transcribed in reverse to form cDNA. RT-qPCR was utilized to measure the expression intensities of *LpBLH3* within these distinct organs [51]. Every experiment was carried out three times. The primer sequences were listed in Table S1.

### 4.4. Subcellular Localization of LpBLH3

The *LpBLH3* gene was inserted into the pBI121-GFP vector by means of specific primers, namely *LpBLH3*-*Bam*HI-F and *LpBLH3*-*Sal*I-R, and then transferred into *Agrobacterium tumefaciens* strain EHA105. Bacterial suspensions were pre-incubated in buffer (10 mM MgCl_2_, 10 mM MES, 200 μM acetosyringone) and injected into four-week-old *Nicotiana tabacum* leaves. After 72 h of dark incubation, the subcellular localization of LpBLH3 was observed using a Carl Zeiss fluorescence microscope (Carl Zeiss, Oberkochen, Germany). The primer sequences were listed in Table S1.

### 4.5. Acquisition of LpBLH3 Overexpressed Lines

The *LpBLH3* gene was integrated into the plant expression vector pCXSN through the utilization of the XcmI restriction enzyme. The verified pCXSN-*LpBLH3* plasmid was introduced into the *Agrobacterium tumefaciens* strain EHA105 (Takara, Tokyo, Japan). Transgenic plants were acquired by means of the *Agrobacterium*-mediated genetic transformation approach. DNA was extracted from the leaves of *LpBLH3* overexpressing *L. pumilum* using the SDS method, and successful transformation was confirmed by PCR, which was conducted using *LpBLH3*-F and *LpBLH3*-R primers. The expression of *LpBLH3* in the overexpressing *L. pumilum* was measured by RT-qPCR using Actin-F/R and FP-F/R primers as controls. The primer sequences were listed in Table S1. Every sample was composed of three biological replicates.

### 4.6. Determination of Physiological Indexes of LpBLH3 OverExperssing L. pumilum

In order to explore how the overexpression of *BLH3* affects the plant’s response to salt stress, we detected and analyzed the physiological indexes. WT and *LpBLH3* overexpressing lines with comparable sizes were cultivated in pots under non-stress conditions. Stress inductions were carried out by irrigating them with 11 mM H_2_O_2_, 200 mM NaCl, 20 mM Na_2_CO_3_, or 20 mM NaHCO_3_ for seven days. The photosynthetic parameters, namely stomatal conductance, transpiration efficiency, net photosynthetic rate, and intercellular CO_2_ concentration, were measured employing an LI-6400 photosynthesis apparatus. The chlorophyll content was quantified using a Chlorophyll Meter SPAD-502Plus (KONICA MINOLTA, Tokyo, Japan). The determination of Malondialdehyde (MDA) content was achieved using the thiobarbituric acid (TBA) approach [52]. The determination of free proline content was accomplished using the ninhydrin protocol. Every sample was composed of three biological replicates.

In order to monitor the alterations in the expression of salt-related genes within *LpBLH3* overexpressing lines, RT-qPCR was employed to determine the expression of *LpSOS1*, *LpNHX1*, *LpABI5*, and *LpMYB4* genes (The primer sequences were listed in Table S1). After subjecting both the *LpBLH3* overexpressing lines and WT lines to a treatment with 20 mM NaHCO_3_ for 24 h, the quantification was carried out in accordance with the previously described approach.

### 4.7. LpBLH3 Regulates the Lignin Content in L. pumilum

Lignin staining was applied to the root and stem of WT and *LpBLH3* overexpressing lines by means of the Wiesner technique [53]. The lignin content determination was carried out with the application of ultraviolet spectrophotometry [54].

In order to verify whether the overexpression of the *LpBLH3* gene in plants will lead to change in the expression of lignin-related genes, we measured and compared the expression levels of the key genes, namely *LpPAL*, *Lp4CL*, *LpC3H*, *LpC4H*, *LpCCoAOMT* and *LpF5H*, in the lignin synthesis pathway (the primer sequences were listed in Table S1).

### 4.8. Screening of LpBLH3 Interacting Protein

The construction of the *L. pumilum* cDNA library was accomplished by OE Biotechnology (Shanghai, China). After that, with the assistance of the EasyGeno Fast Recombination Cloning Kit (TIANGEN, Beijing, China), the *LpBLH3* gene was integrated into pGBKT7 vector through the utilization of the BamHI restriction enzyme. The resultant construct was transformed into the Y2H Gold yeast strain. The screening of the yeast library was carried out in accordance with the guidance provided by Clontech (www.clontech.com, accessed on 7 October 2024). The positive blue colonies were picked out, and PCR was carried out employing the pGADT7 universal primers T7 and 3′-AD. The PCR products were sent to Kumei Biotechnology (Changchun, China) for sequencing analysis. The primer sequences were listed in Table S1. The interacting proteins of BLH3 were predicted by STRING (http://string-db.org/, accessed on 9 October 2024).

### 4.9. Validation of the Interaction Between LpBLH3 and LpKNAT3

Yeast two-hybrid (Y2H) assay was employed to confirm the interaction between LpBLH3 and LpKNAT3. The coding sequence of the candidate interacting protein LpKNAT3 was inserted into the pGADT7 vector with the utilization of *LpKNAT3*-*Eco*RI-F and *LpKNAT3*-*Bam*HI-R primers. The *LpKNAT3*-pGADT7 plasmid was co-transformed with *LpBLH3*-pGBKT7 into the Y2H Gold yeast strain, which was then dropped on SD solid media lacking tryptophan, leucine (SD/-Trp-Leu) and SD/-Trp -Leu -His -Ade + X-α-gal + ABA solid medium, respectively. Co-transformation of pGADT7 and pGBKT7, pGADT7 and pGBKT7-*LpBLH3*, and pGBKT7 and pGADT7-*LpKNAT3* were used as controls. The sequences of the primers were listed in Table S1.

In order to provide further verification for the interaction occurring between LpBLH3 and LpKNAT3, the Luciferase Complementation Imaging (LCI) experiment was implemented. The plasmids, namely *LpBLH3*-pCAMBIA1300-Cluc and *LpKNAT3*-pCAMBIA1300-Nluc (*LpBLH3*-*Kpn*I-F, *LpBLH3*-*Sal*I-R; *LpKNAT3*-*Bam*HI-F, *LpKNAT3*-*Sal*I-R), were transferred into *Agrobacterium tumefaciens* strain GV3101 via the freeze–thaw method [55]. Further, the bacteria were suspended in buffer (10 mM MgCl_2_, 10 mM MES, 200 μM acetosyringone) and incubated for 2 h. Four-week-old *Nicotiana tabacum* leaves were divided into four regions and injected with different bacterial suspensions, which were incubated in the dark for 48 h. The leaves were then injected with d-luciferin potassium salt and incubated in the dark for 5 min. Images were captured using a chemiluminescence imaging system (Tanon-5200, Tanon, Shanghai, China). The primer sequences were listed in Table S1.

### 4.10. Cloning of the LpABI5 Promoter and Analysis

With the aid of a Genome Walking Kit, the promoter sequence of *LpABI5* was cloned (Takara, Tokyo, Japan). The primers (*LpABI5*-SP1, SP2, and SP3) were listed in Table S1. It was subjected to an analysis procedure with the assistance of the PlantCARE (http://bioinformatics.psb.ugent.be/webtools/plantcare/html/, accessed on 9 October 2024). The screened cis-acting elements were visualized and mapped by means of TBtools software V1.0. The complete sequence of the *LpABI5* promoter is presented in Table S3.

### 4.11. Dual-Luciferase Reporter Gene Assay

The *LpABI5* promoter was inserted into the pGreenII0800-LUC vector. The *LpBLH3* and *LpKNAT3* sequence were integrated into the pGreenII62-SK vector with the application of primers *LpBLH3*-*Bam*HI-F and *LpBLH3*-*Xho*I-R. Subsequently, the resultant construct plasmid vectors were transferred into *Agrobacterium tumefaciens* strain GV3101 by the freeze–thaw method. These transformed strains (*LpBLH3*-62SK, *LpKNAT3*-62SK, and *LpABI5*Pro-LUC) were then co-infiltrated into *Nicotiana tabacum* leaves. After 2 days in the dark, the leaves were injected with d-luciferin potassium salt at the injection site and incubated in the dark for 5 min. A chemiluminescent imaging system (Tanon-5200, Tanon, Shanghai, China) was used for imaging. The activities of LUC and REN luciferase were measured using a Dual-luciferase Reporter Gene Assay Kit (Beyotime, Shanghai, China), and LUC activity was normalized to REN activity. The assay was performed in triplicate. The primer sequences were listed in Table S1.

### 4.12. DNA Electrophoretic Mobility Shift Assay (EMSA)

EMSA was carried out to confirm whether LpBLH3 can bind to the *LpABI5* promoter in vitro. This assay was carried out with the use of the BeyoGold™ Chemiluminescent EMSA Kit (Beyotime, Shanghai, China), following the guidelines provided by the manufacturer. The *LpBLH3* gene was cloned into the pET21a vector with a His-tag using the primers *LpBLH3*-*Bam*HI-F and *LpBLH3*-*Sal*I-R, and the recombinant plasmid was transformed into *E. coli* strain BL21. Protein purification was carried out using the BeyoGold™ His-tag Purification Resin Kit (Beyotime, Shanghai, China), following the manufacturer’s instructions. Probes were designed for the LpBLH3 binding sites in the *LpABI5* promoter region (The primers *LpABI5*-TGGA-F and *LpABI5*-TGGA-R were listed in Table S1). The 5′ ends of these probes were labeled with biotin and synthesized by Comate BioScience (Changchun, China). The LpBLH3-His fusion protein was mixed with probes (biotin-labeled, unlabeled, or mutant). After gel electrophoresis, membrane transfer, cross-linking, and other steps, chemiluminescence was used to detect the position of the free and bound protein probes.

### 4.13. Data Statistical Analysis

All treatments were performed randomly and repeated three times. The data underwent statistical analysis with the utilization of SPSS 23.0 software. Statistical significance was regarded as achieved when *p* < 0.05, *p* < 0.01, and *p* < 0.001, which were denoted by *, **, and *** correspondingly.

## Data Availability

Data will be made available on request.

## Supplementary Materials

The following supporting information can be downloaded at: https://www.mdpi.com/article/10.3390/plants14121860/s1, Figure S1: Analysis of the conserved domains of LpBLH3. Figure S2: Amino acid sequence alignment of LpBLH3 protein. Figure S3: LpBLH3 evolutionary tree analysis. Figure S4: Relative expression content of *LpBLH3* in the WT and overexpressing #1–#8 lines. Figure S5: Photosynthetic analysis of *LpBLH3* overexpressing lines under saline-alkaline stress. Figure S6: Verification of LpBLH3 binding to the *LpABI5* promoter by EMSA. Table S1: All primer sequences in this research. Table S2: The sequence of *LpBLH3*. Table S3: The sequence of *LpABI5* promoter. Table S4: Analysis of cis-acting elements of *LpABI5* promoter. Table S5: Candidate interacting proteins obtained by yeast two-hybrid screening of LpBLH3.

## References

[1] Wang J. (2023). How saline-alkaline land transforms into “black soil”: Placing equal emphasis on improvement and cultivation. The People’s Daily.

[2] Yao D., Wu J., Hu Z., Bai B., Zhuang W., Li J., Deng Q. (2019). Physiological mechanisms and breeding strategies for saline-alkali tolerance in rice. Hybrid Rice.

[3] Isayenkov S., Maathuis F. (2019). Plant Salinity Stress: Many Unanswered Questions Remain. Front. Plant Sci..

[4] Gao Y., Zhang J., Wang C., Han K., Hu L., Niu T., Yang Y., Chang Y., Xie J. (2023). Exogenous Proline Enhances Systemic Defense against Salt Stress in Celery by Regulating Photosystem, Phenolic Compounds, and Antioxidant System. Plants.

[5] Guo R., Yang Z., Li F., Yan C., Zhong X., Liu Q., Xia X., Li H., Zhao L. (2015). Comparative metabolic responses and adaptive strategies of wheat (*Triticum aestivum*) to salt and alkali stress. BMC Plant Biol..

[6] Miller G., Suzuki N., Ciftci-Yilmaz S., Mittler R. (2010). Reactive oxygen species homeostasis and signalling during drought and salinity stresses. Plant Cell Environ..

[7] Tan M., Sun S., Wang J. (2023). Bioinformatics and Stress-Responsive Expression Analysis of DREB Transcription Factors in *Lilium pumilum*. J. Northwest For. Univ..

[8] Tao Y., Chen M., Shu Y., Zhu Y., Wang S., Huang L., Yu X., Wang Z., Qian P., Gu W. (2018). Identification and functional characterization of a novel BEL1-like homeobox transcription factor GmBLH4 in soybean. Plant Cell.

[9] Hamant O., Pautot V. (2010). Plant development: A TALE story. Comptes Rendus Biol..

[10] Lee J.-H., Lin H., Joo S., Goodenough U. (2008). Early sexual origins of homeoprotein heterodimerization and evolution of the plant KNOX/BELL family. Cell.

[11] Niu X., Fu D. (2022). The Roles of BLH Transcription Factors in Plant Development and Environmental Response. Int. J. Mol. Sci..

[12] Hao S., Wang Y., Yan Y., Liu Y., Wang J., Chen S. (2021). A Review on Plant Responses to Salt Stress and Their Mechanisms of Salt Resistance. Horticulturae.

[13] Blein T., Hasson A., Laufs P. (2010). Leaf development: What it needs to be complex. Curr. Opin. Plant Biol..

[14] Kumar R., Kushalappa K., Godt D., Pidkowich M.S., Pastorelli S., Hepworth S.R., Haughn G.W. (2007). The Arabidopsis BEL1-LIKE HOMEODOMAIN proteins SAW1 and SAW2 act redundantly to regulate KNOX expression spatially in leaf margins. Plant Cell.

[15] Zhang L., Sun L., Zhang X., Zhang S., Xie D., Liang C., Huang W., Fan L., Fang Y., Chang Y. (2018). OFP1 Interaction with ATH1 Regulates Stem Growth, Flowering Time and Flower Basal Boundary Formation in Arabidopsis. Genes.

[16] Zhang L., Zhang X., Ju H., Chen J., Wang S., Wang H., Zhao Y., Chang Y. (2016). Ovate family protein1 interaction with BLH3 regulates transition timing from vegetative to reproductive phase in Arabidopsis. Biochem. Biophys. Res. Commun..

[17] Zhang J. (2021). Functional Studies of GhBLH5-AO5 and GhHDT4D in Cotton Drought Stress Response. Ph.D. Thesis.

[18] Chen S., Jia Y., Yang Y., Liu H., Chen H., Liu J., Yin H., Zhuo R., Han X. (2025). Genome-wide analysis of the TsBLH gene family reveals TsBLH4 involved the regulation of abiotic stresses by interacting with KNOX6 in *Toona sinensis*. Plant Stress.

[19] Zhao K., Zhang X., Cheng Z., Yao W., Li R., Jiang T., Zhou B. (2019). Comprehensive analysis of the three-amino-acid-loop-extension gene family and its tissue-differential expression in response to saline stress in poplar. Plant Physiol. Biochem..

[20] Liu Y. (2015). Functional analysis of homeodomain transcription factors in secondary cell wall formation in *Arabidopsis thaliana*. Plant Cell.

[21] Chun H.J., Baek D., Cho H.M., Lee S.H., Jin B.J., Yun D.-J., Hong Y.-S., Kim M.C. (2019). Lignin biosynthesis genes play critical roles in the adaptation of *Arabidopsis* plants to high-salt stress. Plant Signal. Behav..

[22] Chen H., Wang J.P., Liu H., Li H., Lin Y.-C.J., Shi R., Yang C., Gao J., Zhou C., Li Q. (2019). Hierarchical transcription factor and chromatin binding network for wood formation in *Populus trichocarpa*. Plant Cell.

[23] Yan C., Hu Z., Nie Z., Li J., Yao X., Yin H. (2021). CcBLH6, a BELl-like homeodomain-containing transcription factor, regulates the fruit lignification pattern. Planta.

[24] Shafi A., Chauhan R., Gill T., Swarnkar M.K., Sreenivasulu Y., Kumar S., Kumar N., Shankar R., Ahuja P.S., Singh A.K. (2015). Expression of SOD and APXB genes positively regulates secondary cell wall biosynthesis and promotes plant growth and yield in *Arabidopsis* under salt stress. Plant Mol. Biol..

[25] Qin W., Yin Q., Chen J., Zhao X., Yue F., He J., Yang L., Liu L., Zeng Q., Lu F. (2020). The class II KNOX transcription factors KNAT3 and KNAT7 synergistically regulate monolignol biosynthesis in *Arabidopsis*. J. Exp. Bot..

[26] Wang S., Yamaguchi M., Grienenberger E., Martone P.T., Samuels A.L., Mansfield S.D. (2020). The Class II KNOX genes KNAT3 and KNAT7 work cooperatively to influence deposition of secondary cell walls that provide mechanical support to arabidopsis stems. Plant J..

[27] Hoth S., Morgante M., Sanchez J.-P., Hanafey M.K., Tingey S.V., Chua N.-H. (2002). Genome-we gene expression profiling in *Arabidopsis thaliana* reveals new targets of abscisic acid and largely impaired gene regulation in the abi1-1 mutant. J. Cell Sci..

[28] Zhang D., Ding X., Wang Z., Li W., Li L., Liu L., Zhou H., Yu J., Zheng C., Wu H. (2025). A C_2_H_2_ Zinc Finger Protein, OsZOS2-19, Modulates ABA Sensitivity and Cold Response in Rice. Plant Cell Physiol..

[29] Kim D., Cho Y., Ryu H., Kim Y., Kim T., Hwang I. (2013). BLH 1 and KNAT 3 modulate ABA responses during germination and early seedling development in *Arabidopsis*. Plant J..

[30] Liu C., Li Z., Dou L., Yuan Y., Zou C., Shang H., Cui L., Xiao G. (2020). A genome-wide identification of the BLH gene family reveals BLH1 involved in cotton fiber development. J. Cotton Res..

[31] Jia T., Wang H., Cui S., Li Z., Shen Y., Li H., Xiao G. (2024). Cotton BLH1 and KNOX6 antagonistically modulate fiber elongation via regulation of linolenic acid biosynthesis. Plant Commun..

[32] Uno Y., Furihata T., Abe H., Yoshida R., Shinozaki K., Yamaguchi-Shinozaki K. (2000). *Arabidopsis* basic leucine zipper transcription factors involved in an abscisic acid-dependent signal transduction pathway under drought and high-salinity conditions. Proc. Natl. Acad. Sci. USA.

[33] Yan F., Deng W., Wang X., Yang C., Li Z. (2012). Maize (*Zea mays* L.) homologue of ABA-insensitive (ABI) 5 gene plays a negative regulatory role in abiotic stresses response. Plant Growth Regul..

[34] Skubacz A., Daszkowska-Golec A., Szarejko I. (2016). The role and regulation of ABI5 (ABA-Insensitive 5) in plant development, abiotic stress responses and phytohormone crosstalk. Front. Plant Sci..

[35] Chang H.-C., Tsai M.-C., Wu S.-S., Chang I.-F. (2019). Regulation of ABI5 expression by ABF3 during salt stress responses in *Arabidopsis thaliana*. Bot. Stud..

[36] Bi C., Ma Y., Wu Z., Yu Y.-T., Liang S., Lu K., Wang X.-F. (2017). *Arabidopsis* ABI5 plays a role in regulating ROS homeostasis by activating CATALASE 1 transcription in seed germination. Plant Mol. Biol..

[37] Bensmihen S., To A., Lambert G., Kroj T., Giraudat J., Parcy F. (2004). Analysis of an activated ABI5 allele using a new selection method for transgenic *Arabidopsis* seeds. FEBS.

[38] Nakashima K., Fujita Y., Katsura K., Maruyama K., Narusaka Y., Seki M., Shinozaki K., Yamaguchi-Shinozaki K. (2006). Transcriptional regulation of ABI3-and ABA-responsive genes including RD29B and RD29A in seeds, germinating embryos, and seedlings of *Arabidopsis*. Plant Mol. Biol..

[39] He H. (2020). Transcriptome analysis of *Lilium pumilum* and Preliminary Exploration of the Function of LpPEX5 and LpPEX7 Genes. Master’s Thesis.

[40] Tadjouri H., Amiri O., Medjedded H., Nemmiche S., Benati F.Z. (2023). Ecophysiological responses of *Glycine max* L. under single and combined cadmium and salinity stresses. Ecotoxicology.

[41] Li Z., Su X., Chen Y., Fan X., He L., Guo J., Wang Y., Yang Q. (2022). Melatonin Improves Drought Resistance in Maize Seedlings by Enhancing the Antioxidant System and Regulating Abscisic Acid Metabolism to Maintain Stomatal Opening Under PEG-Induced Drought. J. Plant Biol..

[42] Zhang J., Li H., Huang X., Xing J., Yao J., Yin T., Jiang J., Wang P., Xu B. (2022). STAYGREEN-mediated chlorophyll a catabolism is critical for photosystem stability during heat-induced leaf senescence in perennial ryegrass. Plant Cell Environ..

[43] Chen H., Wu W., Du K., Ling A., Kang X. (2018). The interplay of growth-regulating factor 5 and BZR1 in coregulating chlorophyll degradation in poplar. Plant Cell Environ..

[44] Hu P., Zhang K., Yang C. (2019). BpNAC012 positively regulates abiotic stress responses and secondary wall biosynthesis. Plant Physiol..

[45] Yang Q., Yuan C., Cong T., Wang J., Zhang Q. (2022). Genome-wide identification of three-amino-acid-loop-extension gene family and their expression profile under hormone and abiotic stress treatments during stem development of Prunus mume. Front. Plant Sci..

[46] Zhao H. (2020). The Molecular Mechanisms of Plant Responses to salt stress. Front. Plant Sci..

[47] Nookaraju A., Pandey S.K., Ahlawat Y.K., Joshi C.P. (2022). Understanding the modus operandi of Class II KNOX transcription factors in secondary cell wall biosynthesis. Plants.

[48] Collin A., Daszkowska-Golec A., Szarejko I. (2021). Updates on the Role of ABSCISIC ACID INSENSITIVE 5 (ABI5) and ABSCISIC ACID-RESPONSIVE ELEMENT BINDING FACTORs (ABFs) in ABA Signaling in Different Developmental Stages in Plants. Cells.

[49] Zhang J., Gai M., Xue B., Jia N., Wang C., Wang J., Sun H. (2017). The use of miRNAs as reference genes for miRNA expression normalization during Lilium somatic embryogenesis by real-time reverse transcription PCR analysis. Plant Cell.

[50] Liang S., Liu Y.Y., Lin N.F. (2013). Saline-alkaline Grassland Improvement through the Bio-engineering Technology. Adv. Mater. Res..

[51] Zhang L., Wang Z., Ji S., Zhu G., Dong Y., Li J., Jing Y., Jin S. (2024). Ferric reduction oxidase in *Lilium pumilum* affects plant saline-alkaline tolerance by regulating ROS homeostasis. Plant Physiol. Biochem..

[52] Senthilkumar M., Amaresan N., Sankaranarayanan A. (2021). Estimation of Malondialdehyde (MDA) by Thiobarbituric Acid (TBA) Assay. Plant-Microbe Interactions.

[53] Xu L., Zhu L., Tu L., Liu L., Yuan D., Jin L., Long L., Zhang X. (2011). Lignin metabolism has a central role in the resistance of cotton to the wilt fungus *Verticillium dahliae* as revealed by Rna-Seq-dependent transcriptional analysis and histochemistry. J. Exp. Bot..

[54] Xie X.-M., Zhang X.-Q., Dong Z.-X., Guo H.-R. (2011). Dynamic changes of lignin contents of MT-1 elephant grass and its closely related cultivars. Biomass Bioenergy.

[55] Ge Q. (2005). Cloning of the Choline Monooxygenase Gene of Salix Salina and Its Expression in Tobacco. Master’s Thesis.

