# Supplementary material for: BLH3 Regulates the ABA Pathway and Lignin Synthesis Under Salt Stress in Lilium pumilum
Source: Plants (Basel). 2025 Jun 17;14(12):1860. doi: 10.3390/plants14121860 (PMC12196856; doi:10.3390/plants14121860)
Supplement: Supplementary file 1 [file plants-14-01860-s001.zip › plants-3691150-supplementary.pdf]

Table S1. All primer sequences used in this study.

LpBLH3-F	ATTCCCTAAGATCCTGTTCTAATG
LpBLH3-R	TCAATTCATCTGAATTCTCTGATTCCC
*LpBLH3-Bam*HIF	GGATCCATGGAAAATGATATGTTTAATG
*LpBLH3-Sal*IR	GTCGACATTCATCTGAATTCTCTG
LpBLH3-SP1	GAATCAGGTATCTCACCATTGGCCA
LpBLH3-SP2	GCCAACCATAATCTGCCCGTAGT
LpBLH3-SP3	TGGTCTGACATCGGTGGCAGAA
qPCR-*LpBLH3*-F	AGTACCACAGCTGCAGACAC
qPCR-*LpBLH3*-R	ACAGATTTCTCAGGCAGGCC
*LpBLH3-Bam*HI-F	GGATCCATGGAAAATGATATGTTTAATG
*LpBLH3-Xho*I-R	CTCGAGTCAATTCATCTGAATTCTCTG
*LpBLH3-Sal*I-F	GTCGACATGGAAAATGATATGTTTAATG
*LpBLH3-Kpn*I-R	GGTACCATTCATCTGAATTCTCTGAT
*LpBLH3-Sma*I-F	CCCGGGATGGAAAATGATATGTTTAAT
*LpBLH3-Sal*I-R	GTCGACTCAATTCATCTGAATTCTCTG
*LpBLH3-Sal*I-R-TGA	GTCGACATTCATCTGAATTCTCTG
*LpBLH3-Not*I-R	GCGGCCGCTCAATTCATCTGAATTCT
*LpBLH3-Sfi*I-F	GGCCNNNNNGGCCATGGAAAATGATATGTTT
LpBLH3-SalI-1300F	TGCAGGGGCCCGGGGTCGACATGGAAAATGATATGTTTAATG
*LpBLH3-Sal*I-1300R-TGA	ACTAGTATTTAAATGTCGACCATTCATCTGAATTCTCTGATT
LpBLH3-1100F	AAAAGCCTCACCTCTCCC
LpBLH3-1100R	TAGAACAGGATCTTAGGGAATTAC
LpBLH3-1036F	CCTACTCCTCTGTCCGAGC
LpABI5-SP1	AGAACAGAATCATGGGCTAGTTTCCC
LpABI5-SP2	GGGTAGGCAAGGATAGGGTTTGTGAT
LpABI5-SP3	CCATGTACTGTGATGAAGGGCTTTGT
LpKNAT3-F	ATGGAGATGGGAGGGATTG
LpKNAT3-R	TTACCTTTTGCGCTTAGACT
*LpKNAT3-EcoR*I-F	GAATTCATGGAGATGGGAGGGATTGC
*LpKNAT3-Bam*HI-R	GGATCCTTACCTTTTGCGCTTAGACT
*LpKNAT3-Xba*I-F	TCTAGAATGGAGATGGGAGG
*LpKNAT3-Sal*IR-TGA+A	GTCGACACCTTTTGCGCTTAG
*LpKNAT3-Bam*HI-F	GGATCCATGGAGATGGGAGGGA
*LpKNAT3-Xho*I-R	CTCGAGTTACCTTTTGCGCTTAGACT
3’-AD	AGATGGTGCACGATGCACAG
T7	TAATACGACTCACTATAGGG
LpSOS1-qpcr-F	GCGATCCGTTGATGAGGCTA
LpSOS1-qpcr-R	CTACACGGAGGACCTGAGGA
LpNHX1-qpcr-F	GTGCGACTGCTATGTGCAAG
LpNHX1-qpcr-R	CGCTTTGTTCAGTTGGCGAT
LpABI5-qpcr-F	ACGGTGGCGGTATGATGTTT
LpABI5-qpcr-R	TCCCAAACTAGCCGGAGTA
LpMYB4-qpcr-F	AGAAGACGACCGGCTTATCG
LpMYB4-qpcr-R	CCGGTCACAGCTTGATGAGT
LpABI5 - TGGA - F	TGAAAACATGGACGATTATATACCTCCATTTGTCAAATTG
LpABI5 - TGGA - R	CAATTTGACAAATGGAGGTATATAATCGTCCATGTTTTCA

Lp4CL-qpcr-F	ATGAAAGGCTATCTC
Lp4CL-qpcr-R	TCAACCGTCAATCCG
LpF5H-qpcr-F	ATGCTGATCTATAATC
LpF5H-qpcr-R	TTACGGCGCCCATTGC
LpC4H-qpcr-F	ATGGTAATGACTTCCG
LpC4H-qpcr-R	CTAACTTATCCTGCC
LpCCOAOMT-qpcr-F	ATGGAAGAGATACAA
LpCCOAOMT-qpcr-R	CTAAAGTAATGGATAACG
LpPAL-qpcr-F	ATGAACATGGGGACCG
LpPAL-qpcr-R	TACCACCTGCACCAAC
LpHCT-qpcr-F	ATGATCATCAATGTGAGGG
LpHCT-qpcr-R	CTACATATCATAGAGCAAC
Lp4CL-qpcr-F	ATGAAAGGCTATCTC
Lp4CL-qpcr-R	TCAACCGTCAATCCG
LpF5H-qpcr-F	ATGCTGATCTATAATC
LpF5H-qpcr-R	TTACGGCGCCCATTGC
LpCAT1-qpcr-*F*	GTGCCGGTTTGGTTCTTGTC
LpCAT1-qpcr-*R*	CGCATCGATCGACCCTAACA
LpATEM-qpcr-*F*	TGGCAGGAGCCATTGTTAGG
LpATEM-qpcr-*R*	ACACCCACGTCCCAATCTTC
LpRD29B-qpcr-*F*	GACTTCACCCCCTTAGGCAC
LpRD29B-qpcr-*R*	AAGCCGACGTAAGCGAAGAA
LpABF3-qpcr-*F*	CACTGTTCAATGGAGCGCAG
LpABF3-qpcr-*R*	GCGGCTGACTCCCTATTCTT

Table S2. The sequence of *LpBLH3*

ATGGAAAATGATATGTTTAATGTTCTGCCACCGATGTCAGACCATAACCATATGGTCTTTGAC

GCAGTCTCGCCACATATGTTTTCAAGTCCGTTCTTTCAGTCGGGCATACATGATTACAACAA
CTACGGGCAGATTATGGTTGGCGAGCCCTTGTTTCCAACTTTCCAAGACGAAACTCTGAAC
AATCAGTTTATGGCAAACAATGGTGTTATGGCCAATGGTGAGATACCTGATTCTAGAAATAT
GCCTCATGTTGATTGTACTTCATTTAGCAACTCCATCGGAAGTGCCAATCTCATGGGAAATTC
CTTATCGGCCACTTCTCTTGCACATCTTTTGTCCTCGTCTACCACTTTGCAGGATAATCGCCA
CAATAGTGGCATTGCTTCTAATCCCGCAGCACTGAGGAACTCTCTGACTAGCGATAGCTGTA
GCACTTTAAATTCATCTCTTACAGCCTCGGTAAACTGCGAATTCGGTCCACACGAAGATACC
GATATTCTAACCGAGAAAAAGAGCAACTCAAGCGAGCAACCAAACTATAGATGGAGCTACG
ATGAATTTCTCGGCTATCAGGTGCCTTCAACTAGAAGTATAAGCATGGTTCGTCCATCCTATC
ATGTCATCGGAAGCTTGGAGCCTGGATGGAGTTCCGATAGGTCAATTTTGAACATTGACCAT
CCATACAGTTTTTGTGGGGCTAGTAACGAGCTATCGTTGAGTCTTGGTTCCTGCCAGCCTTC
ATTGATCAACATGCCTAATGTTCCAGACCAGTGCTCAGAAGTCAGCTGCTCCGGCCTATCGC
AGGTCACATCAAAAGATAATAGATACCCGCAATCTATTGATATGCCAGTCTTTCAGAACTCTT
TGCACTGTGAGGAGCTTTCTTTGCACTGTGGATCTTCTAGCCAGTCGAACTTTCCTCGTGTT
GTATTAGGATCGAAATATCATCGTGTAGCCCAAGAAATACTTGCTGGAATTGCCAACTATGC
ACTAGAGGAAGTAGATGAGATGGATGATTCAGTAGGCGGGGTTGAGACCGAGGCAAACAT
GTCTTTTTCTTCTCATCGGCACGGTCTGAAAGGATTCACGTTATCAGGAAGCGACGATTTTC
CATGTTCTACGGGAGAAAGCAAGTCTCATGGCTCGATTGATCCCCCGCACCAACGGGAGGA
AAATTATTCAAAGAAATCTGAGCTGTTAACTATGCTTCAAATGGTCGATAAGAAATACAATC
AGTGCTTGGACCAGATGCAGCATGCCATATGTTCATACGAAAGTACCACAGCTGCAGACAC
ACCTCCAATGCTTGCCCGTTTTGCCGTTCATAGCATCTCCGTTCTGTACAAAAACTTGAGGG
AGAGTATTGCCAGTCAGATTGTTATGATCAATCAGCATCCTGGTGGTGAACATACAGGAGAA
AAAGATAGAACCTTTGAAACATCGTTTATCAGAAAACAATGGGCTTTGCAACAGCTGAGAA
GGAACGAGCAACAATCTTGGAGGCCTCAGAGGGGCCTGCCTGAGAAATCTGTCTCGGTTCT
ACGTGCATGGATGTTCCAAAACTTTCTTCACCCGTACCCAAAAGACAATGAAAAGCATTTG
CTAGCCATTCAAAGTGGATTGACGAGAAGCCAGGTGTCCAACTGGTTTATAAATGCGCGCG
TTCGCCTCTGGAAACCATTGATAGAGGAAATGTACTCGGAGGTAAACAAAAAGAATCGTCC
GATGGAAGGATCAGGTTGCGATAGCAGGAACTATGCAAGCATTGGGAATCAGAGAATTCAG
ATGAATTGA

Table S3. The sequence of *LpABI5* promoter.

CTATAGGGCACGCGTGGTTGACGAGAGAAATGAACAACAATCTGATTGTCTCATAAATCTTC<br>ATCACTTCGATATCCCGTGCGCACCAACTGCTCTCTTTACTTGTTGTTAGACTAACAGAGTAT<br>TTAGAAGTAGTGAAGCTGATACATACCAACGTCATCTAACATACCTTACAACCATATCGCCTC<br>CTTCACTTCTTAAGTAACCGGCGACTATGTATGGTTGCAGTAGATTGTATGGAATGTTGGTAT<br>AGCGGAGGAAGTGAAGAATTCATTGGCCGACGTATCGACCTTTATCGTCGATAAAGCCACTA<br>TGGATTGCAAGATATAAAAAATATGCCCACTTGTCAATGCATAGCTGGAAATAGCAGCTATTT<br>CGGTGATACCTAACGTTCTATATTTTTTATACGGGTGAACAGTTTAAGTCCCATTTATTCTTGT<br>GAAAACATGACCGATTATATACCTCCATTTGTCAAATTGTCCAGCAAAATATTCAGGGTAAAT<br>AAGAACACTTTTGTACTGGCTAATATATGGAGGTAAACAGTTTAACAGGTCGTTTTACAAAA<br>TCAATAACTTCTCGCCTAAAGAAGAGACGGTGGGAGTATATCCCAAGAAAACTTGGGCTGG<br>CATAGTTTTAGTTATTGAAGAGCGGATTTCTTCTCTGCCACCCTCATATAGGGTTCTTTTGAA<br>CCCGACCGTATGGAACTTTGTTTCAAGGACAAGTGGCTAACACGAATAACTAAAATTTATAG<br>GGTGTTCACGTAATTAACCCCTTGAAACAAAGTTCCTGTTCACCGATTGTGCTTATTGATTTT<br>AAATATCCCACAAGTGCATTAATTGGAGCCATCATCACTCAGCTCCACCAATATTGGCTCCC<br>AAGAGTAATCAAATTGAGTGTAAGATTAGACAAATCGGTAGTAGTGAGTCGAGGTGGTTAT<br>AACCGAGGGTTCTCATTAGTTTAACTCACATTCTAATCTGTTTCCCATGCTCATAACGTGTCA<br>GAACGAGCCTATCCAACGGCCCACGATGGCCACGCTCTCCTTGCTGGGTACGAGGGTGATT<br>GCACAGTCTTGCTCGGATAGGTTGCCGGGTGCTGACCGGTGCGGAGAGGAACGCATTAGTC<br>CACCCATCCCGTCAACCACCGCTCCCCTCAAAATCTCCATCTCGCCGGCGACCGACACCTG<br>GTAATCAGGTGGGTAGGGCAGTTGGTGGCGAAGGGGAGTTTTAGAGGTAGAGCGGCCGCT<br>GGCTGTGGACTCCAGCCGCAATCGCCACGCGCCGCCTGCTCACCAGATCGCCAACAGGTCC<br>ATTCCTCTGCTCTTTCTATAGGTCGGCGTTAGCGGTGCGCGGCGGACGAGTGGTCTAGCGGT<br>TGTCCAGGTAAGGAGACGAGAAAGATATCTCGGCTTCATATTTCGCTGGATTTGACTCCGAA<br>GCAATAAATTGATGTGAACTTGATCTACTTCAGATAGAGCCGAAGTATAAAGCGACCTAAAC<br>TGAGGCTTCGTTATTTAACTACACTTGAACTAGATGAAGTCTACTCGGAATTAGAGTTTATGA<br>TTTTGTGATGAGCACTTCTAGTTGTTGATTCTACAAATTTTTCTATAGATGAGCCTTAATCTCA<br>AATACTAAAACACTACTCGTGAAGATCAACAACTAAGATGTTTAAAAAGATATCTAGAACTA<br>CAAGCTTGATGTTC

Table S4. Analysis of cisacting elements of *LpABI5* promoter

Functional element	number	coresequence	function
TATAbox	13	TATA/ TATAA/ ATTATA/ TACAAAA/ CCTATAAAAA	Core promoter element around 30 of transcription start
CAATbox	17	CAAT/ CAAAT/ CCAAT	Commoncis acting element in promoter and enhancer regions
ABRE	5	ACGTG/ GACACGTGGC	cisactingregulatoryelementessentialfortheanaerobicinduction
AuxRRcore	1	GGTCCAT	Involved in regulating the auxin response elements
CATbox	2	GCCACT	Cisacting regulatory elements associated with meristem expression
CGTCAmotif	3	CGTCA	Cisacting regulatory element involved in the MeJA responsiveness
TGACGmotif	3	TGACG	Cisacting regulatory element involved in the MeJA responsiveness
GBox	4	CACGTT/ TACGTG/ CACGAC	Light responsive element
GT1motif	1	GGTTAAT/ GGTTAA	Light responsive element
TCTmotif	1	TCTTAC	Light responsive element
chsCMA1a	1	TCTTAC	Light responsive element
TATCbox	1	TATCCCA	Cisacting elements involved in the gibberell in reactivity
MYB	8	CAACCA/ CAACAG/ CAACTG/ CCGTTG/ TAACCA	The MYB protein binding site
MYC	1	CATTTG	The MYC protein binding site
BLH3like（BLH）	8	TGAC	The BLH3 protein binding site

Table S5. Candidate interacting proteins obtained by yeast two-hybrid screening of LpBLH3

Number	Gene name.	Gene annotation.
One	STR16	Encodes rhodanese-like sulfurtransferase.
Two	PITH	PITH Structural protein.
Three	RHC1A	Encodes the E3 ubiquitin ligase GA receptor GID1.
Four	RHA1B	RING-H2 structure E3 ubiquitin ligase.
Five	PYR6	Encodes uridine 5'-monophosphate (UMP)/cytidine 5'-monophosphate (CMP) kinase.
Six	ACO2	Encodes aconitate hydratase.
Seven	MAH1	Encodes alkane hydroxylase.
Eight	HAT14	Homeodomain-leucine zipper protein.
Nine	KNAT3	A member of the class II KNOX family.
Ten	PGLR	Encodes a cell wall-localized polygalacturonase.

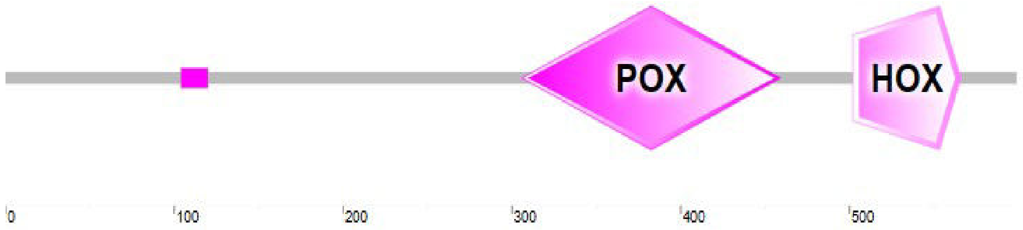

Figure S1. Analysis of the conserved domains of LpBLH3. BLH3 contains one conserved POX domain and one conserved HOX domains, Specifically, the POX domain is located between amino acids 306-459, while the HOX (HD) domain is located between amino acids 502-566.

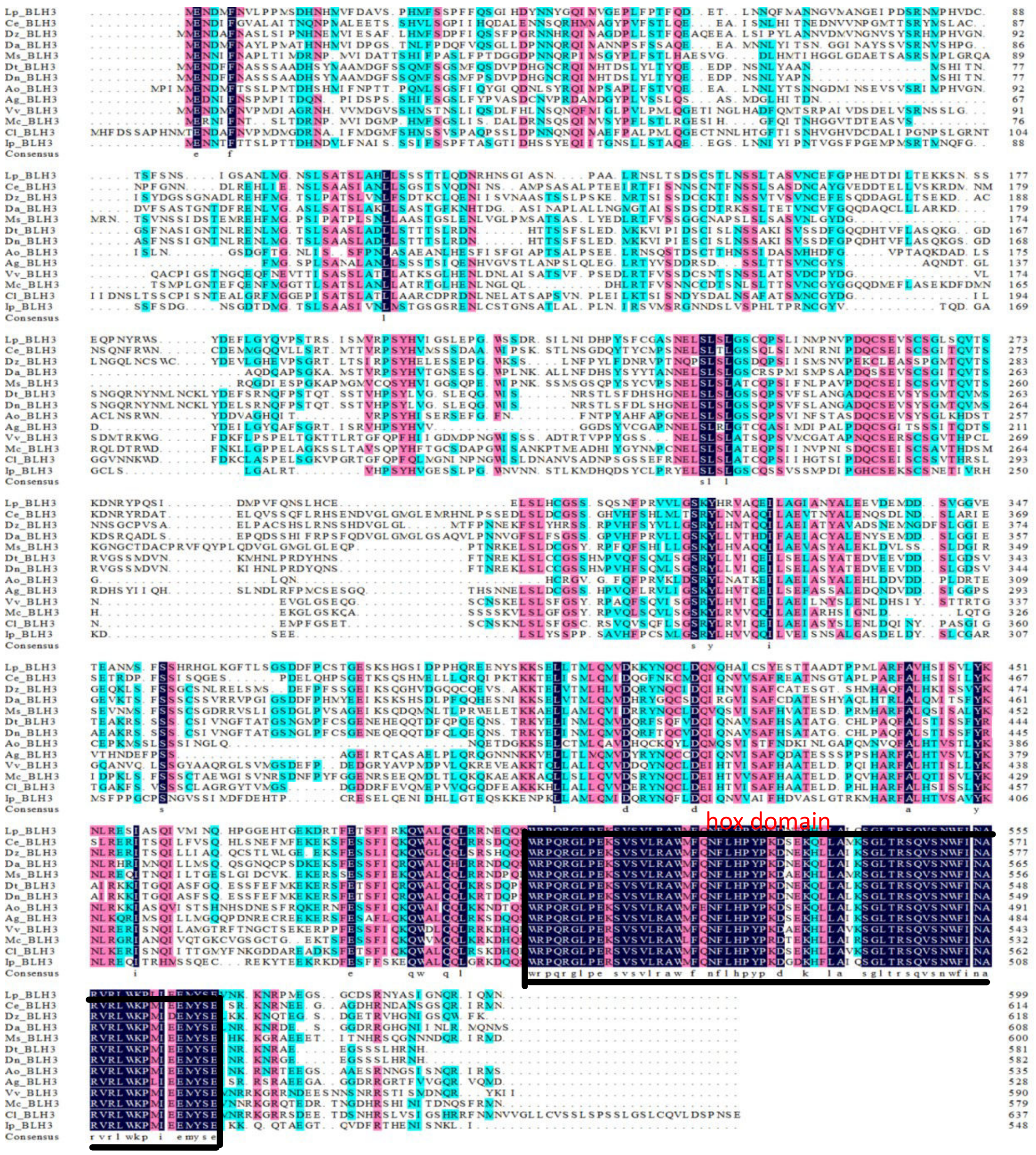

Figure S2. Amino acid sequence alignment of LpBLH3 protein. Amino acid sequence alignment of LpBLH3 with homologous BLH3 proteins from various plant species showed high similarity in sequence. Homology levels were represented as follows: 100% homology is indicated in black, ≥ 75% in pink, and ≥ 50% in cyan on the graph. The species included in the alignment are as follows: *Lp* (*L. pumilum*), *Dn* (*Dendrobium nobile*, KAI0488079.1), *Dt* (*Dendrobium thyrsiflorum*, KAL0904037.1), *Eg* (*Elaeis guineensis*, XP_010915332.1), *Ao* (*Asparagus officinalis*, XP_020249125.1), *Dz* (*Dioscorea zingiberensis*, KAJ0964617.1), *Da* (*Dioscorea alata*, KAH7657571.1), *Ce* (*Colocasia esculenta*, MQL84505.1), *Ag* (*Acorus gramineus*, KAK1267312.1), *Ms* (*Magnolia sinica*, XP_058074064.1), *Mc* (*Macleaya cordata*, OVA11348.1), *Vv* (*Vitis vinifera*, XP_010648330.1), *Cl* (*Camellia lanceoleosa*, KAI7989227.1), *Ip* (*Iris pallida*, KAJ6823017.1).

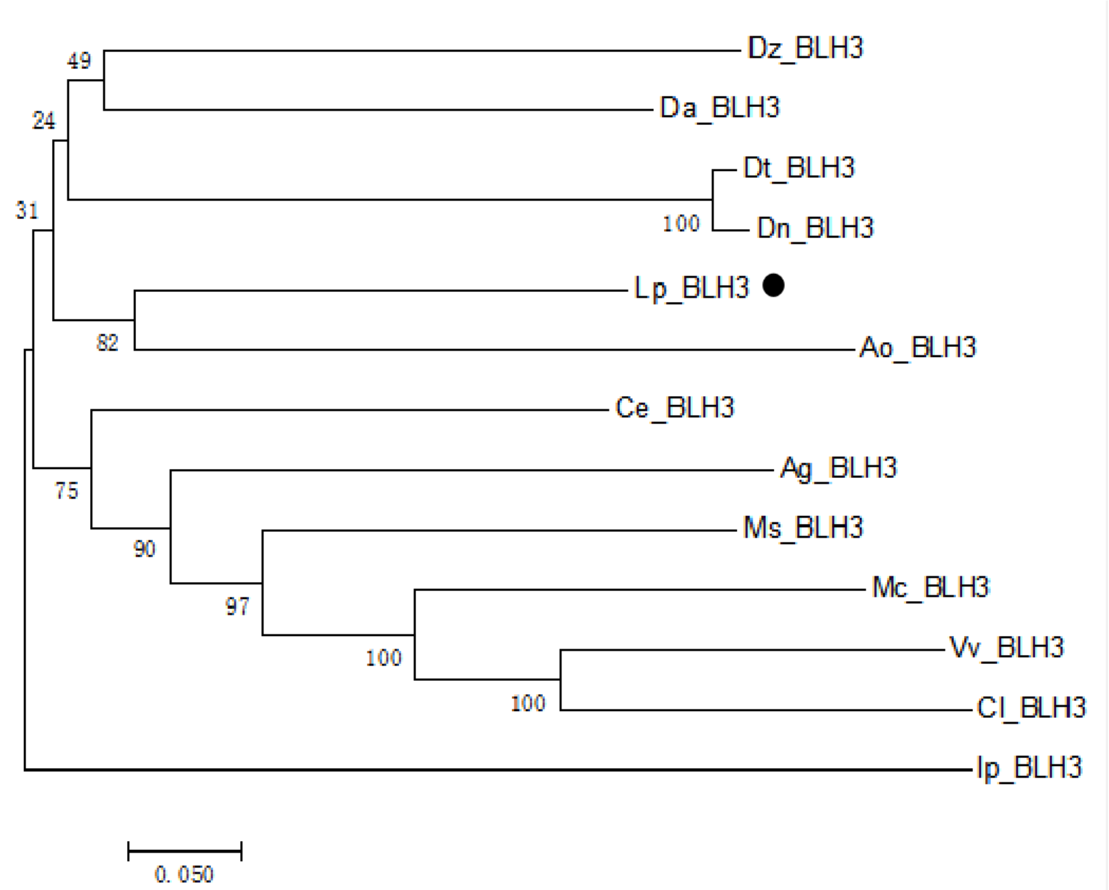

Figure S3. LpBLH3 evolutionary tree analysis. A phylogenetic tree based on the amino acid sequences of LpBLH3 and other plant BLH3 proteins was constructed using MEGA7.0 software. Sequence alignment was performed with the ClustalW algorithm, and the Neighbor-Joining method was used to build the tree. The bar indicates 0.015 substitutions per amino acid position. The scale bar represents a 5% difference in sequence length.

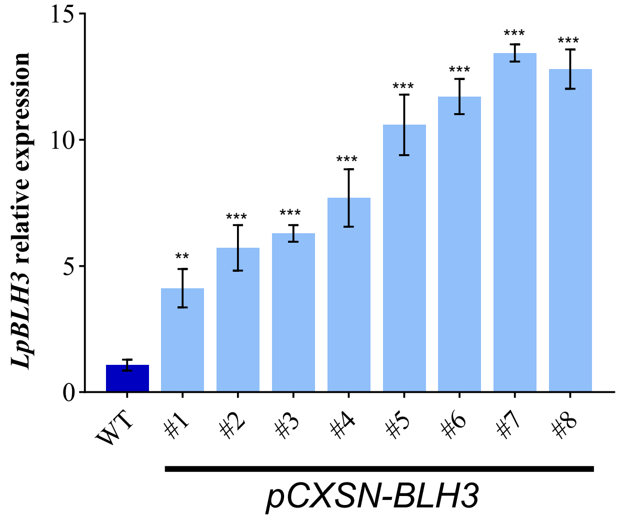

Figure S4. Relative expression content of *LpBLH3* in the WT and overexpressing 1-8 lines. The expression levels of *LpBLH3* were detected by RT-qPCR in *LpBLH3*-overexpressing lines 1-8, which showed higher expression levels of *LpBLH3* compared to the wild-type (WT). Asterisks (**) and (***) indicate statistically significant differences at $p < 0.01$ and $p < 0.001$, respectively. Data are presented as mean ± SD from three replicates.

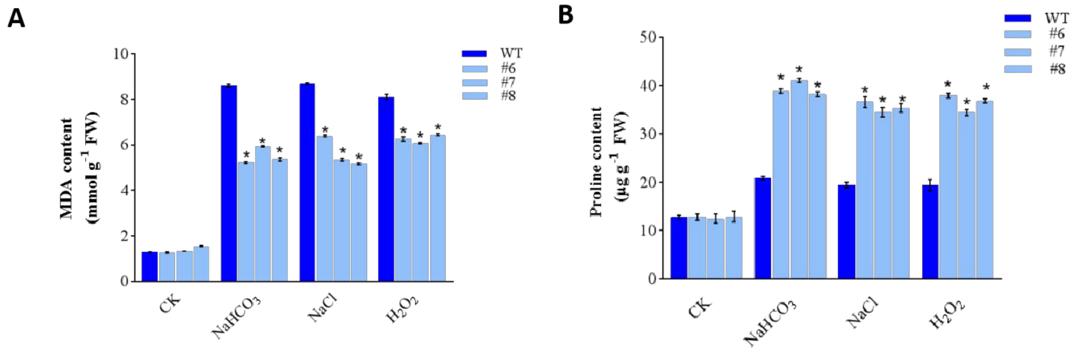

Figure S5. Determination of physiological indexes of *LpBLH3* overexperssing *L. pumilum* under saline stress. (**A**): MDA content. (**B**): Proline content. WT: wild type. #6; #7, #8: *LpBLH3* overexpressing lines. Three selected *LpBLH3* overexperssing lines with high expression level. *P<0.05 standard error of three biological replicates.

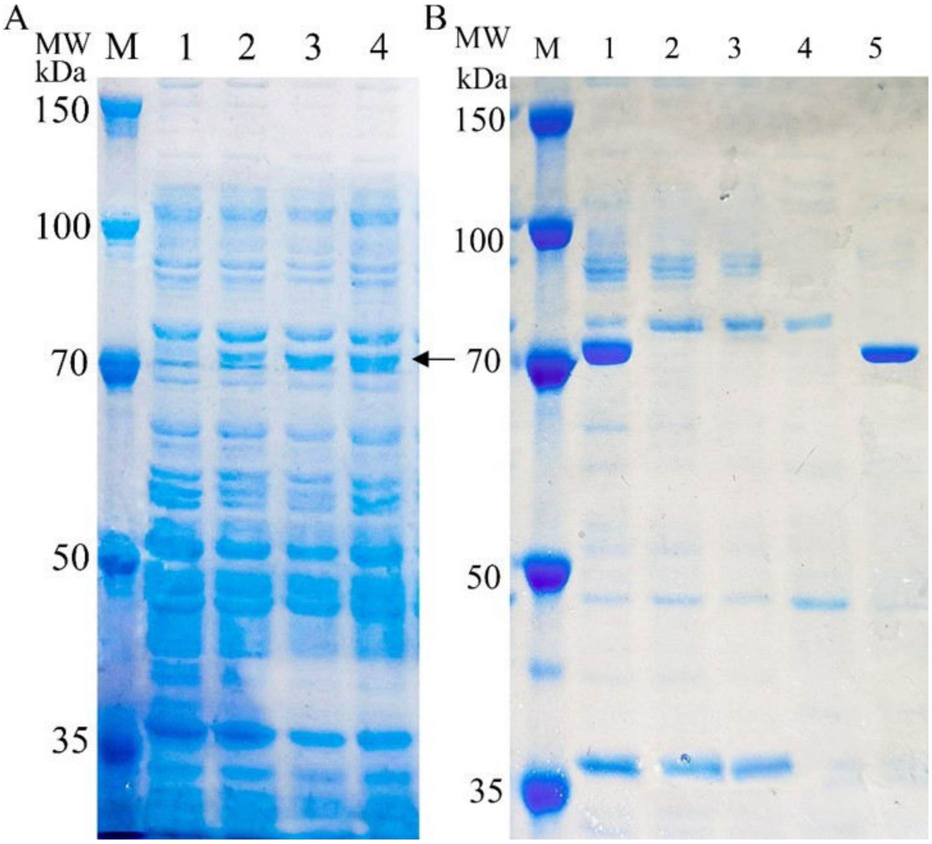

Fig. S6. Verification of LpBLH3 bind to the *LpABI5* promoter by EMSA assay.

(**A**): pET21a-LpBLH3 protein induction by different time. M: protein marker. 1, 2, 3, 4: the expression of LpBLH3 protein was induced by 0h, 1h, 3h and 4h.

(**B**): pET21a(+)-LpBLH3 protein purification. M: protein marker. 1: pET21a-LpBLH3 protein supernatant. 2-4: cleaning solution 1-3. 5: pET21a-LpBLH3 purified protein.
